# Recombination between Homologous Chromosomes Induced by Unrepaired UV-Generated DNA Damage Requires Mus81p and Is Suppressed by Mms2p

**DOI:** 10.1371/journal.pgen.1005026

**Published:** 2015-03-04

**Authors:** Yi Yin, Thomas D. Petes

**Affiliations:** Department of Molecular Genetics and Microbiology and University Program in Genetics and Genomics, Duke University Medical Center, Durham, North Carolina, United States of America; CABIMER, Universidad de Sevilla, SPAIN

## Abstract

DNA lesions caused by UV radiation are highly recombinogenic. In wild-type cells, the recombinogenic effect of UV partially reflects the processing of UV-induced pyrimidine dimers into DNA gaps or breaks by the enzymes of the nucleotide excision repair (NER) pathway. In this study, we show that unprocessed pyrimidine dimers also potently induce recombination between homologs. In NER-deficient *rad14* diploid strains, we demonstrate that unexcised pyrimidine dimers stimulate crossovers, noncrossovers, and break-induced replication events. The same dose of UV is about six-fold more recombinogenic in a repair-deficient strain than in a repair-proficient strain. We also examined the roles of several genes involved in the processing of UV-induced damage in NER-deficient cells. We found that the resolvase Mus81p is required for most of the UV-induced inter-homolog recombination events. This requirement likely reflects the Mus81p-associated cleavage of dimer-blocked replication forks. The error-free post-replication repair pathway mediated by Mms2p suppresses dimer-induced recombination between homologs, possibly by channeling replication-blocking lesions into recombination between sister chromatids.

## Author Summary

Ultraviolet (UV) light is a ubiquitous agent of exogenous DNA damage. In normal cells, the nucleotide excision repair (NER) pathway is the primary mechanism for repair of UV-induced DNA lesions. Defects in the NER pathway are associated with the human disease xeroderma pigmentosum (XP), and XP patients are prone to skin cancer. Mitotic recombination is strongly stimulated by UV treatment. In this study, we examined whether such stimulation requires the NER pathway. We show that, in the absence of NER, UV is still able to greatly induce recombination. We then characterized a nuclease that is required to generate recombinogenic breaks. Finally, we examined a previously known recombinogenic pathway called the “post-replication repair (PRR) pathway.” Our results suggest that the PRR pathway mainly promotes recombination between sister chromatids, and suppresses recombination between chromosome homologs.

## Introduction

The primary types of DNA lesions caused by UV radiation are pyrimidine dimers [1]. Although UV strongly stimulates recombination in wild-type yeast cells [2–5], it is unclear whether this stimulation in wild-type cells primarily reflects unexcised dimers, or single-stranded DNA gaps and double-stranded DNA breaks (DSBs) resulting from incomplete nucleotide excision repair (NER) of dimers. One approach to simplifying the nature of the recombinogenic lesion is to examine UV-induced recombination events in NER-deficient cells. Previously, Kadyk and Hartwell (1993, [6]) showed that NER-deficient *rad1* strains had reduced levels of UV-induced inter-homolog recombination and elevated levels of sister chromatid recombination compared to UV-induced events in an NER-proficient strain. Based on these observations, they argued that the inter-homolog recombinogenic effects of UV in wild-type cells were likely a consequence of DNA lesions introduced during NER. Since Rad1p can process various types of secondary DNA structures [7,8], one caveat to this conclusion is that Rad1p could be involved in the downstream events of recombination in addition to its role in producing the recombinogenic lesion. Consequently, in the current study, we examined UV-induced recombination in a *rad14* diploid. Strains that lack Rad14p cannot perform NER, but are not known to have any other recombination defect [9]. Our analysis demonstrates that inter-homolog recombination events of a variety of types (crossovers, gene conversions unassociated with crossovers, and break-induced recombination events) are greatly elevated by unexcised dimers. Our analysis is the first detailed study of the recombinogenic effects of this biologically-important DNA lesion.

Unexcised pyrimidine dimers block or slow the progression of replication forks [10]. In yeast, G1-synchronized *rad14* mutants treated with UV have large (up to 3000 base) single-stranded regions on the leading strand of the replication fork, as well as smaller single-stranded gaps on both the leading and lagging strands [11]. In addition, these strains have asymmetric fork structures diagnostic of broken forks. Presumably, either the single-stranded regions or the DSBs resulting from broken forks could act as recombinogenic lesions. Regressed replication forks are not observed as a consequence of unrepaired UV damage in yeast [11], although evidence for such structures has been obtained in *E*. *coli* [10].

**Fig 1:**
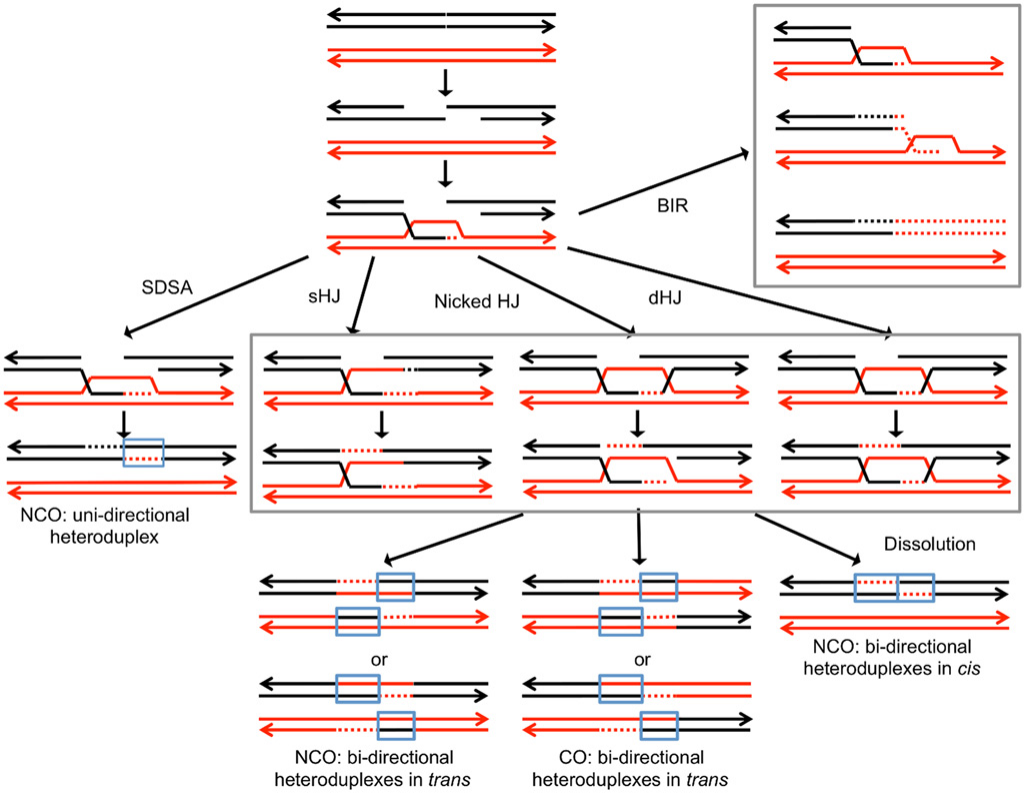
Pathways of homologous recombination. We depict recombination events initiated by a double-stranded DNA break (DSB) with each chromosome shown as a double-stranded DNA molecule. All pathways are initiated by the invasion of the 3’ end of the broken DNA molecule. Dotted lines denote DNA synthesis primed by the invading end. Regions in which a red strand and black strand are paired (in blue boxes) represent heteroduplexes; repair of mismatches within the heteroduplexes can generate gene conversion events. In synthesis-dependent strand annealing (SDSA), following DNA synthesis primed from the invading strand, the invading strand is displaced, hybridizing to the other broken end. This pathway produces a gene conversion that is not associated with a crossover (NCO). In Holliday junction (HJ) containing intermediates, an association of both broken ends with the intact template molecule can result in formation of a single Holliday junction (sHJ), a nicked Holliday junction, or a double Holliday junction (dHJ). Cleavage of these junctions by resolvases can produce either non-crossovers (NCO) or crossovers (CO). For both CO and NCO products, both molecules contain heteroduplexes located in *trans*. In contrast, if the HJ is resolved by dissolution, the heteroduplex regions are located in *cis*. In break-induced replication (BIR) events, the invading end copies the intact homolog by conservative DNA replication, resulting in a large terminal LOH event.

Broken DNA molecules can be repaired by a variety of homologous recombination (HR) pathways involving the intact homolog (Fig. 1). Gene conversion events unassociated with crossovers occur primarily through synthesis-dependent strand-annealing (SDSA), although a small fraction are a consequence of processing of a double-Holliday junction (dHJ) or dHJ dissolution [8,12]. In addition, a broken DNA end can invade a duplex, generating a replication structure that duplicates sequences extending to the terminus of the homolog. This process, termed “break-induced replication” (BIR), involves conservative DNA replication [13,14]. These pathways generate a variety of branched intermediates including nicked HJs, double HJs, and single HJs that must be resolved to produce mature recombinant products. Processing of intermediates may involve either cleavage or dissolution (Fig. 1).

For the cleavage of various types of HJs formed during mitotic HR in *S*. *cerevisiae*, the primary resolvase is the Mus81p/Mms4p complex with Yen1p serving as a backup role [15]. The Mus81p/Mms4p complex prefers nicked HJs as a substrate, whereas Yen1p more efficiently cleaves intact junctions [8]. The nucleases involved in cleavage of HJs are somewhat organism specific and/or specific to the type of recombination (meiotic versus mitotic). For example, Rad1p has no role in junction resolution during meiotic recombination in yeast [16], but its ortholog in Drosophila (MEI-9) is required for meiotic recombination [17]. We show below that recombination between homologs caused by unexcised dimers requires Mus81p but not Yen1.

In addition to DNA repair events that involve inter-homolog interactions, DNA lesions that stall replication forks can be bypassed by both error-free and error-prone pathways of post-replication repair (PRR; [18]). The error-free pathway utilizes HR, primarily operating within the context of the replication fork, to bypass DNA damage. One sub-pathway involves template switching between the two arms of the replication fork, whereas a second pathway involves fork regression. Since fork regression is not evident for UV-induced damage [11], we assume that the primary error-free pathway for UV-induced damage in yeast is template switching. Efficient template switching depends upon the poly-ubiquitination of PCNA by the Ubc13p/Mms2p complex [19]. In contrast, the error-prone pathway utilizes the error-prone translesion synthesis (TLS) DNA polymerases to replicate the damaged DNA strand [18]. The recruitment of TLS polymerases requires mono-ubiquitinated PCNA catalyzed by the Rad6 and Rad18 proteins and is not known to be recombinogenic [19]. In our analysis, we examined strains lacking Mms2p, since such strains should be defective specifically in the error-free component of PRR. We find that *mms2* strains have elevated levels of inter-homolog recombination, indicating that the Mms2p suppresses inter-homolog recombination.

## Results

### Detection of inter-homolog recombination and mapping of loss of heterozygosity events

**Fig 2:**
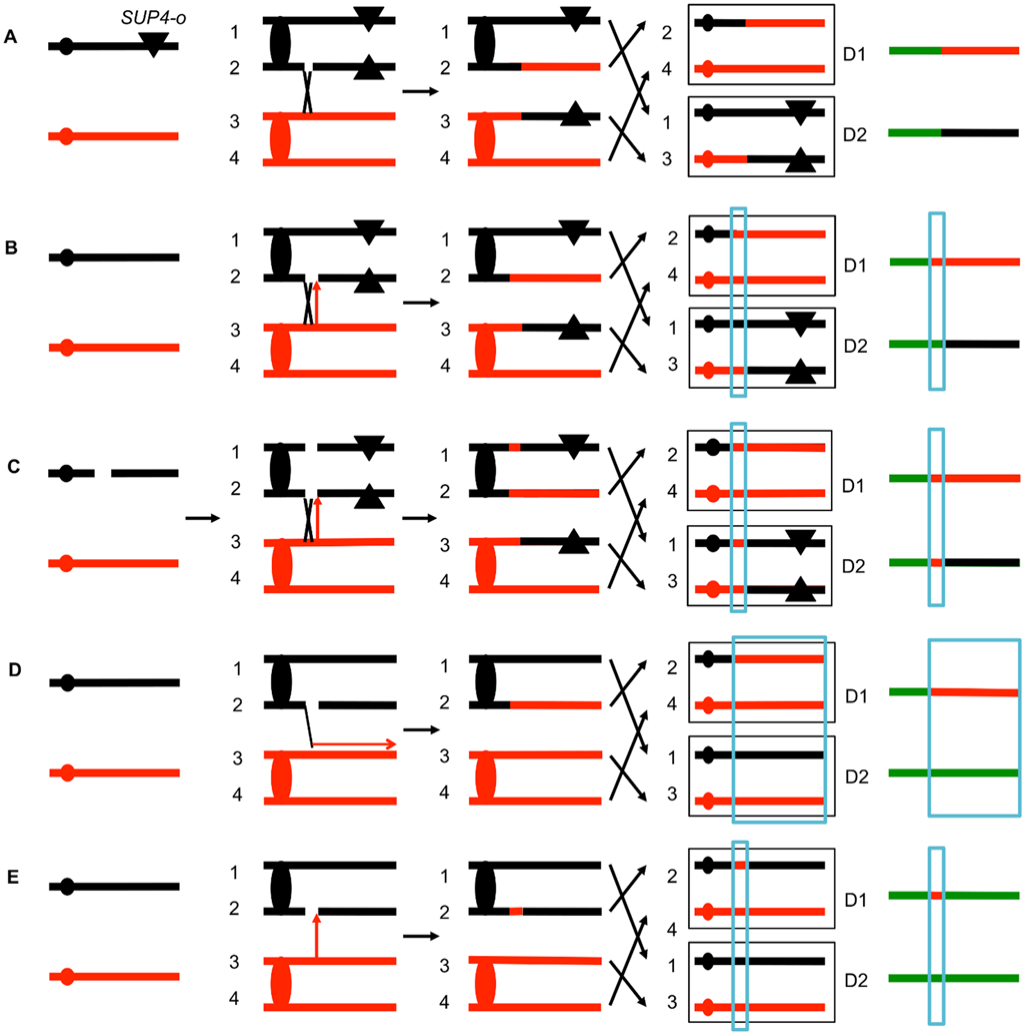
Patterns of loss of heterozygosity (LOH). As described in the text, diploids were derived from two sequence-diverged haploids. The diploids were homozygous for the ochre suppressible *ade2–1* mutation and heterozygous for the ochre suppressor *SUP4-o* tRNA (shown as a triangle) near the left end of chromosome V or the right end of IV. Zero, one, and two copies of *SUP4-o* in the diploid produce red, pink or white colonies, respectively. The black and red lines indicate the homolog derived from the haploids YJM789 and W303–1A, respectively. The boxed chromosomes on the right side of the Fig represent recombinant products. Next to these products, we show the patterns of heterozygous markers and homozygous markers (identified by SNP arrays) as lines. Chromosome regions that are heterozygous, homozygous for W303–1A-derived SNPs, or homozygous for YJM789-derived SNPs are represented by green, red, and black segments, respectively. D1 and D2 indicate the LOH patterns in the two daughter cells that contain the recombinant products. A. Simple crossover. When the red and white sectors are examined by SNP arrays, for this type of recombination event, D1 and D2 are identical in the positions of the transition between heterozygous and homozygous SNPs. This result is expected for a crossover without an associated gene conversion. B. Crossover with a 3:1 conversion tract. In this example, when the red and white sectors are examined, the transition positions between heterozygous and homozygous SNPs are not identical. In the region that is boxed in blue, considering both sectors, three of the chromosomes have “red” SNPs and only one has “black” SNPs. C. Crossover with a 4:0 conversion. In this red/white sectored colony, there is a region adjacent to the crossover in which all four chromosomes have the “red” SNP. We interpret this pattern as resulting from the repair of two sister chromatids that are broken at the same position. It is likely that these events are a consequence of a DSB in an unreplicated chromosome that is subsequently replicated. Repair of one of the broken chromatids is associated with a conversion and a crossover, whereas the second chromatid is repaired by a conversion event unassociated with a crossover. D. BIR event. A DSB on the black chromatid is repaired by a BIR event that duplicates sequences from the red chromatid. Note that this event can be detected on any of the chromosomes, not just the one marked with the *SUP4-o* gene. E. Conversion event unassociated with a crossover. As noted in Fig. 2D, because of the high frequency of LOH events in UV-treated cells we can detect classes A to E as unselected events.

The system that we used to detect and map UV-induced mitotic recombination events has been described previously [3,4,20]. In brief, we constructed a diploid heterozygous for about 55,000 heterozygous single-nucleotide polymorphisms (SNPs) by crossing two haploids (W303–1A and YJM789) that had 0.5% sequence divergence. In addition, the diploid was heterozygous for an insertion of the ochre suppressor tRNA *SUP4-o* located near the left end of chromosome V and homozygous for the *ade2–1* ochre suppressible mutation. The starting diploid with one copy of *SUP4-o* partially suppresses the red pigment that accumulates in *ade2* strains. Following a mitotic crossover, if the recombined chromosomes are segregated into different daughter cells, one daughter cell would lack the suppressor and one cell would have two copies of *SUP4-o* (Fig. 2A). Continued divisions of each of these cells would result in a red/white sectored colony with red sector derived from the daughter cell with no copies of the suppressor and the white sector derived from the daughter cell with two *SUP4-o* genes.

The position of the crossover is the junction between heterozygous SNPs and homozygous SNPs. Mapping of the position of loss of heterozygosity (LOH) was done using oligonucleotide-based microarrays [3]. For about 13,000 SNPs distributed throughout the genome, these arrays contained short (25-base) oligonucleotides that were specific to the W303–1A form of the SNP or the YJM789-specific form. By comparing the level of hybridization of the genomic samples with the recombination event to a heterozygous control strain (details in Materials and Methods), we could determine the transition between heterozygous SNPs and homozygous SNPs.

Several different patterns of LOH were observed. In some red/white sectored colonies, the positions of the transition were identical in both sectors (Fig. 2A). The microarray analysis for the two homologs is depicted as a single line for each sector with heterozygous chromosomal regions shown in green, and regions homozygous for W303–1A-derived SNPs and YJM789-derived SNPs shown as red and black segments, respectively. There were two other types of red/white sector colonies that were commonly observed. As shown in Fig. 2B, some of the sectored colonies had a chromosome segment (boxed in blue), adjacent to the crossover that was not reciprocally transferred between chromosomes. These segments are gene conversion events and likely reflect DNA mismatch repair within a heteroduplex tract adjacent to the crossover (Fig. 1). About 80–90% of mitotic crossovers are accompanied by a gene conversion [4,20]. Considering both chromosomes in both sectors, the conversion event shown in Fig. 2B has three chromosomes with W303–1A-derived SNPs and only one chromosome with YJM789-derived SNPs. Such events will be called “3:1” conversions, and these events are a consequence of repair of a single chromatid break (SCB) as shown by the arrow in Fig. 2B. In previous studies of spontaneous and UV-induced mitotic recombination [4,20,21], we also found red/white sectors in which the conversion event produced a 4:0 conversion associated with the crossover. This pattern is a consequence of the repair of double sister chromatid breaks (DSCB) at the same position. Our favored interpretation of this result [22] is that a chromosome that received a break in G1 was replicated to produce two broken sister chromatids (Fig. 2C).

**Fig 3:**
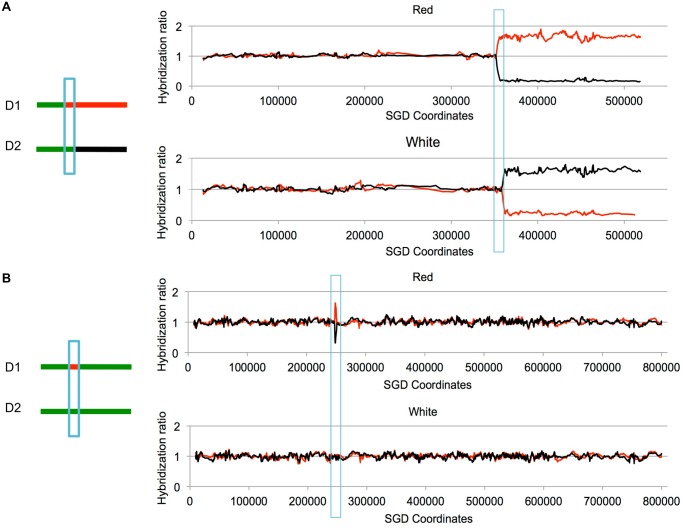
Examples of LOH events as detected by SNP microarrays. Cells were treated with UV and allowed to form colonies. We isolated DNA from the red and white sectors of sectored colonies, and examined the samples using SNP microarrays. Although these sectors were selected to have a reciprocal crossover on chromosome V, the two events shown below are unselected events on other chromosomes. Experimental samples were hybridized in competition with a differentially-labeled heterozygous control sample. On the Y-axis is shown the relative hybridization level of the experimental and control samples to SNP-specific oligonucleotides. Red and black colors indicate hybridization to W303–1A-specific and YJM789-specific SNPs, respectively. A value of 1 indicates heterozygosity. The X-axis shows SGD coordinates for the individual chromosomes. A. Unselected crossover on chromosome VIII. On chromosome VIII, there is a transition to homozygosity of red SNPs in the red sector and to homozygosity of black SNPs in the white sector. The positions of the transition are not identical in the sectors, indicating a 3:1 gene conversion (blue box) associated with the crossover. B. Unselected gene conversion event on chromosome II. In the red sector, there is a small interstitial LOH region. The white sector has no LOH events. This pattern would be produced by a gene conversion event unassociated with a crossover, or by a conversion event associated with a crossover in which the two recombinant chromosomes co-segregate.

Other patterns of LOH can be detected as unselected events by microarrays. For example, BIR can produce one daughter cell with a terminal LOH event and a second daughter cell that has no LOH (Fig. 2D). In addition, unselected gene conversion events that are unassociated with crossovers can produce one daughter cell with an interstitial LOH event and a second daughter cell with no LOH (Fig. 2E). Examples of LOH patterns diagnostic of a conversion-associated crossover, and a conversion unassociated with a crossover are shown in Fig. 3.

In most of the experiments described below, yeast strains with the *SUP4-o* marker located near the left end of chromosome V or near the right end of chromosome IV were synchronized in G1 and treated with UV. All diploids used in our study had deletions of the *MATα* gene, allowing their synchronization with *α* pheromone [22]. The cells were plated on solid growth medium, immediately treated with UV, and subsequently screened for sectored colonies. Cultures derived from each side of the sector were examined by microarrays. For most of the experiments, we used arrays that allowed us to monitor LOH events on the selected chromosome (either V or IV) and unselected events throughout the genome.

### Unexcised dimers strongly stimulate mitotic recombination in UV-treated G1-synchronized cells

The NER-deficient *rad14* mutant is very sensitive to UV radiation [9]. While 15 J/m^2^ of UV in G1-arrested wild-type diploids did not significantly reduce cell viability in the wild-type (76% viability relative to unirradiated cells; [4]), less than 0.02% of the *rad 14* cells survived 5 J/m^2^ of UV. Treatment of *rad14* cells with 1 J/m^2^ of UV resulted in 61% cell viability, comparable to the viability of wild-type cells treated with 15 J/m^2^. Since formation of a sectored colony requires the survival of both daughter cells containing the products of recombination, it is important that the UV dose has a minimal effect on viability of the treated cells. Previously, we mapped UV-induced LOH in both 1 J/m^2^- and 15 J/m^2^-treated wild-type cells [4]. Below, we show sectoring frequency, as well as the frequency of unselected LOH events, in the *rad14* mutant treated at 1 J/m^2^, in wild-type cells treated at 1 J/m^2^ (resulting in the same number of UV-induced lesions), and in wild-type cells treated with 15 J/m^2^ (an approximately equitoxic radiation dose).

The sectoring frequency data for diploids with the *SUP4-o* marker on either chromosome V or on IV are summarized in Table 1. Since only half of crossovers have the segregation pattern that produces reciprocal LOH in the daughter cells [23], the rate of mitotic crossovers is twice the frequency of red/white sectored colonies for all strains. For most strains treated with UV, the sector frequencies were determined non-selectively. For the untreated wild-type strain with the *SUP4-o* marker on chromosome V, crossovers were selected as described by Lee *et al*. [21]. This method, which involves selection of crossovers as canavanine-resistant sectored colonies, did not work well for strains with the *rad14* mutation or for strains with the *SUP4-o* marker on chromosome IV [20]. In the *rad14* mutant treated with 1J/ m^2^ of UV, the red/white sectoring frequency on the left arm of chromosome V is 0.5%; this frequency is similar to that of the wild-type strain treated with 15 J/m^2^, and is five-fold higher (p = 1.7×10^-10^) than the wild-type treated with 1 J/m^2^ [4] as shown in Table 1. For the same interval, the spontaneous sectoring frequency for the wild-type strain is 1.10.5×10^-6^. In the wild-type strain, we can select crossovers on the left arm of V as canavanine-resistant red/white sectors [21,24]. For unknown reasons, this selection does not work in the *rad14* diploids and, therefore, we screened for sectored colonies. Since no such colonies were detected in 22797 total colonies (Table 1), we conclude that the frequency of spontaneous crossovers in *rad14* strains is very low, less than 4.4 ×10^-5^.

**Table 1:** Frequency of sectored colonies in unirradiated and UV-irradiated wild-type and *rad14* strains.^1^

Strain name	Strain genotype	Location of *SUP4-o*	UV dose (J/m^2^)	# red/white sectored colonies/total colonies	Freq. of red/white colonies (95% CI)	Freq. of red/white and pink/red/white colonies (95% CI)^6^	Fold increase red/white colonies
PG311^2^	WT	V	0	NR^3^	1.1x10^-6^ (0.6–1.6x10^-6^)	NR^3^	1
PG311^4^	WT	V	1	47/45544	1.0x10^-3^ (0.7–1.3x10^-3^)	1.6x10^-3^ (1.2–2.0x10^-3^)	938
PG311^4^	WT	V	15	36/7194	5.0x10^-3^ (3.6–7.0x10^-3^)	9.5x10^-3^ (7.4–12x10^-3^)	4549
JSC25^5^	WT	IV	0	55/1761664	3.1x10^-5^ (2.4–4.1x10^-5^)	7.9x10^-5^ (6.7–9.3x10^-5^)	1
JSC25	WT	IV	1	18/2564	7.0x10^-3^ (4.4–11x10^-3^)	11x10^-3^ (6–16x10^-3^)	225
JSC25^4^	WT	IV	15	110/1420	7.7x10^-2^ (6.5–9.3x10^-2^)	8.9x10^-2^ (7.5–11x10^-2^)	2481
YYy23.4	*rad14*	V	0	0/22797	<4.4x10^-5^	4.4x10^-5^ (2.2–280x10^-6^)	1
YYy23.4	*rad14*	V	1	23/4853	4.7x10^-3^ (3.1–7.1x10^-3^)	10x10^-3^ (7.5–13x10^-3^)	>108^7^
YYy37.6 YYy37.8	*rad14*	IV	0	20/144771	1.4x10^-4^ (0.9–2.2x10^-4^)	4.5x10^-4^ (3.5–5.8x10^-4^)	1
YYy37.6 YYy37.8	*rad14*	IV	1	99/3216	3.1x10^-2^ (2.5–3.7x10^-2^)	6.5x10^-2^ (5.7–7.4x10^-2^)	223^8^

^1^As discussed in the text, red/white sectored colonies result from mitotic crossovers between *SUP4-o* and the centromere. The diploids used in our analysis had *SUP4-o* located near the left end of chromosome V or the right end of chromosome IV.

^2^Data from Lee *et al*. (2009).

^3^The selective method used in Lee *et al*. (2009) prevented the growth of cells without a recombination event on chromosome V.

^4^Data from Yin and Petes (2013). For the samples irradiated with 15 J/m^2^, we included only those samples grown in the absence of canavanine, and incubated at 30^0^ following irradiation.

^5^Data from St. Charles and Petes (2013).

^6^As described in the text, most of the pink/red/white colonies in the wild-type strains reflect the plating of two adjacent cells, one with a crossover and one with unassociated events.

^7^Relative to the unirradiated wild-type strain PG311, the increase is 4309-fold.

^8^Relative to the unirradiated wild-type strain JSC25, the increase is 986-fold. Note that the unirradiated *rad14* strains YYy37.6 and YYy37.8 are hyper-Rec.

In order to obtain a more accurate estimate of the frequency of sectored colonies in the unirradiated *rad14* strain, we examined sectored colonies in strains in which the *SUP4-o* marker was located on the right arm of chromosome IV (Table 1). The distance between *CEN4* and *SUP4-o* is 1.1 Mb, about eight times larger than the interval between *CEN5* and *SUP4-o* in the other strains. Sectored colony formation in the *rad14* mutant was stimulated about 200-fold by the 1 J/m^2^ dose of UV relative to the unirradiated *rad14* strain. The frequency of red/white sectored colonies in *rad14* strains treated with 1 J/m^2^ UV dose was about four-fold higher than the frequency of such colonies in the wild-type strain treated with the same dose. Interestingly, the *rad14* mutation resulted in a modest (about five-fold, p = 1.8×10^-9^) increase in the frequency of sectors in the absence of UV irradiation.

In addition to the frequencies of red/white sectors, we also show in Table 1 the frequencies of sectors that were either red/white or pink/red/white. Pink/red/white sectors could arise in two different ways: 1) persistence of DNA damage beyond the first division cycle following UV treatment (S1 Fig.), or 2) irradiation of two adjacent cells resulting in a recombination event in one of them (the red/white portion of the sector) but no recombination event in the other (the pink portion of the sector). By micromanipulation of individual cells before irradiation, we showed that most (9 out of 10) of the selected crossovers occur in the first cell division in UV-treated wild-type strains [4], therefore, the pink/red/white sectored colonies we recovered from plating experiments are more consistent with the second explanation.

The recombinogenic effects of unexcised UV-induced DNA damage were further demonstrated by our whole-genome analysis of sectored colonies derived from UV-treated wild-type and *rad14* strains (Table 2). This analysis allows the detection of unselected crossovers, gene conversions, and BIR events throughout the genome. The *rad14* strain treated with 1 J/m^2^ had about seven unselected LOH events per sectored colony, whereas the wild-type strain treated with the same dose had only 0.4 LOH events per sectored colony. The unselected events from the wild-type strain treated with 15 J/m^2^ are described in S1 Table and S2 Table, whereas the unselected events from the *rad14* strain are described in S3 Table and S4 Table. S1 Table and S3 Table depict the transitions between heterozygous and LOH regions within each sector schematically, and S2 Table and S4 Table show the SGD coordinates for each transition. Comparable data for unselected events for the wild-type strain irradiated with 1 J/m^2^ were described previously [4], as were the methods for depicting various classes of LOH events [3,4]. S5–S13 Tables summarize the transitions for the other UV-treated mutant strains, and S14 Table and S15 Table show the genome regions covered by the SNP arrays.

**Table 2:** Numbers of unselected UV-induced LOH events, deletions/duplications, and chromosome number changes in sectored colonies derived from UV-treated wild-type, *rad14*, *mus81*, and *yen1* diploids.^1^

Strain name	Genotype	UV dose (J/m^2^)	# Interstitial LOH events (ave. #/sector)	# Crossovers (ave. #/sector)	# BIR events^2^ (ave. #/sector)	Total LOH events (ave. #/sector)	# Sectors analyzed	# Insertion/deletions	# Ploidy changes
PG311	WT	1	3 (0.3)	1 (0.1)	0 (0.0)	4 (0.4)	10	0	0
PG311	WT	15	141 (7.1)	50 (2.5)	10 (0.5)	201 (10.1)	20	0	0
YYy23.4	*rad14*	1	47 (3.9)^3^ ^,^ ^4^	18 (1.5)^5^	16 (1.3)	81 (6.7)^3^	12	4	0
YYy76.14	*mus81*	15	54 (7.7)	7 (1.0)^4^	6 (0.9)	67 (9.6)	7	0	1
YYy71.28 YYy71.48	*yen1*	15	68 (5.2)	28 (2.2)	3 (0.2)	99 (7.6)	13	1	0

^1^For all strains, we examined unselected LOH events and other genomic changes in sectored colonies resulting from crossovers on chromosome V.

^2^We operationally define a BIR event as a terminal LOH event that is present in one sector but not the other. As discussed in the text, since we purify single colonies from each sector, we cannot exclude the possibility that some of the putative BIR events are actually crossovers that occur in the second division after irradiation.

^3^ p<0.001 compared to wild-type cells treated at 1 J/m^2^.

^4^ p<0.01 compared to wild-type cells treated at 15 J/m^2^.

^5^ p<0.01 compared to wild-type cells treated at 1 J/m^2^.

For statistical comparisons of the number of unselected recombination events for the wild-type and *rad14* strains irradiated with 1 J/m^2^, we determined the number of LOH events per sectored colony for each strain (ten and twelve sectored colonies from the wild-type and *rad14* strains, respectively; Table 2), and compared these distributions by the Mann-Whitney test. By this test, the number of unselected events per colony for the *rad14* strain (average of 6.7) was significantly greater (p = 0.0003) than the number observed for the wild-type strain (average of 0.4).

### Qualitative comparison of UV-induced recombination events in *rad14* and wild-type strains

As shown in Fig. 2, the patterns of LOH in a sectored colony can reveal whether a mitotic recombination event is a consequence of the repair of a DSB on a SCB or repair of a DSCB. 3:1 conversion tracts (Fig. 2B and Fig. 2E) are interpreted as SCB events, whereas 4:0 tracts (Fig. 2C) are indicative of DSCBs [4,21]; crossovers without a detectable conversion tract cannot be classified as SCB or DSCB. We found previously that high doses of UV (15 J/m^2^) in G1-synchronized wild-type strains result in more DSCBs than SCBs, but low doses (1 J/m^2^) produce primarily SCBs [4]. In the G1-synchronized *rad14* strain treated with 1 J/m^2^, 78% of the selected events were SCBs, similar to the fraction observed for the wild-type strain treated with the same UV dose. The data from both the selected events on chromosome V and the unselected events on other chromosomes derived from sectored colonies are in Table 3. In summary, our results demonstrate that UV-induced recombination events in *rad14* strains usually involve a single broken sister chromatid.

**Table 3:** Numbers of single-chromatid breaks (SCBs) and double sister-chromatid breaks (DSCBs) induced by UV in G1-synchronized cells.

Strain name	Genotype	UV dose (J/m^2^)	# selected SCBs^1^	# selected DSCBs^1^	# unselected SCBs^2^	# unselected DSCBs^2^	Total # SCBs (%)	Total # DSCBs (%)
PG311	WT^3^	1	31	10	NA	NA	31 (76%)	10 (24%)
PG311	WT^3^	15	5	12	76	106	92 (44%)	118 (56%)
YYy23.4	*rad14*	1	7	2	58	18	65 (76%)	20 (24%)^4^
YYy76.14	*mus81*	15	2	5	27	38	29 (40%)	43 (60%)
YYy71.28 YYy71.48	*yen1*	15	2	10	46	48	48 (45%)	58 (55%)

^1^Sectored colonies resulting from a crossover on the left arm of chromosome V were examined by microarrays. Based on the LOH patterns in the sectors, events were classified as a SCB (usually diagnosed by a 3:1 conversion associated with the crossover) or a DSCB event (usually diagnosed by a 4:0 or 3:1/4:0 hybrid event). Crossovers without detectable gene conversion events were excluded from classification as SCB or DSCB.

^2^Unselected events are LOH events other than those on the left arm of chromosome V. These events were detected using whole-genome SNP arrays.

^3^These data are a sub-set of those described previously (Yin and Petes, 2013).

^4^ p<0.0001 compared to wild-type cells treated at 15 J/m^2^.

The *rad14* strain irradiated with 1 J/m^2^ had significantly different proportions of recombination events relative to the wild-type strain irradiated with an equitoxic UV dose (15 J/m^2^) (Table 2). More specifically, the *rad14* strain had significant elevations in the number of BIR events and the number of deletions relative to the wild-type strain (p = 0.0003 and 0.0004, respectively). Interestingly, we found presence of early/middle-firing ARS sequences [25] within the deleted sequences in all the 4 deletion events. Although no significant differences were observed between the *rad14* and wild-type strains irradiated with 1 J/m^2^, there were very few unselected LOH events in the wild-type strain.

In considering the frequency of BIR events in the *rad14* strain, we note an important caveat. BIR events in red/white sectored colonies are defined as unselected non-reciprocal terminal LOH events (S2 Fig.). If UV-irradiated *rad14* strains retain recombinogenic lesions beyond the first division cycle, this same LOH pattern could be produced by having one UV-induced crossover on chromosome V in the first division cycle (resulting in the red/white sectored colony) and a second UV-induced LOH event on an unselected chromosome arm in the second division cycle (S3 Fig.). The second LOH event would produce two different types of granddaughters (shown as GD2–1 and GD2–2 in S3 Fig.). In our analysis of sectored colonies, we purify single colonies derived from each sector. If we examined one colony derived from the white sector (either GD1–1 or GD1–2) and a colony derived from GD2–1 from the red sector, we would conclude that there was a reciprocal crossover on chromosome V and an unselected BIR event on the other homolog. In conclusion, the relative frequency of unselected crossovers and BIR events in the UV-irradiated *rad14* strain is unclear. This same caveat applies to all genetic analyses in which the experimental strain has high levels of on-going genetic instability.

To clarify whether unrepaired UV-induced lesions in *rad14* strains were capable of generating recombination events after the first cell division, we micromanipulated single G1-arrested *rad14* cells to specific positions on solid medium that we then irradiated. Of 1096 irradiated (1 J/m^2^) *rad14* cells, 681 cells survived. Of four resulting sectored colonies, two were red/white as expected for a crossover on chromosome V in the first division, and two were pink/red/white as expected for a crossover in the second division (S1 Fig.). The same micromanipulation analysis for the wild-type strain treated with 15 J/m^2^ yielded nine colonies with red/white sectors and only one colony with pink/red/white sectors [4]. Although the numbers of these events were small and the difference in proportions of red/white and pink/red/white sectors in wild-type and *rad14* cells is not statistically significant (p = 0.18 by Fisher exact test), these results suggest that recombinogenic DNA damage can persist in *rad14* cells, as expected from unexcised pyrimidine dimers.

### Recombination caused by unexcised dimers is stimulated by Mus81p but not by Yen1p

Mus81p is the main Holliday junction resolvase for mitotic crossovers in *S*.*cerevisae* [15] and for meiotic crossovers in *S*. *pombe* [26]. In addition, *mus81* strains are UV sensitive [27]. To determine whether Mus81p had a role in the processing of UV-induced recombinogenic lesions, we examined the frequency and types of UV-induced recombination in a *mus81* strain and in a *mus81 rad14* double mutant strain.

For the *mus81* strain, we used microarrays to perform a genome-wide analysis of seven red/white sectored colonies derived from G1-synchronized cells irradiated with 15 J/m^2^. There was no significant difference (p = 0.89 by Mann-Whitney test) in the average number of interstitial LOH events (primarily gene conversions) in the *mus81* strain compared to wild-type (7.7 and 7.1, respectively). In contrast, the average number of unselected crossovers in the *mus81* strain was reduced to 40% of the level observed in the wild-type strain (Table 2). This loss of crossovers is statistically significant (p = 0.004 by Mann-Whitney test). These results are similar to those observed for HO-induced DSBs by Ho *et al*. [15], demonstrating that Mus81p was involved in the resolution of HJs by crossovers, but not in the SDSA conversion pathway. In addition, the single *mus81* mutant does not significantly (p = 0.72 by Mann-Whitney test) reduce the frequency of recombination events, since the average number of LOH events per sectored colony is very similar for the wild-type and *mus81* strains (10.1 and 9.6, respectively).

**Fig 4:**
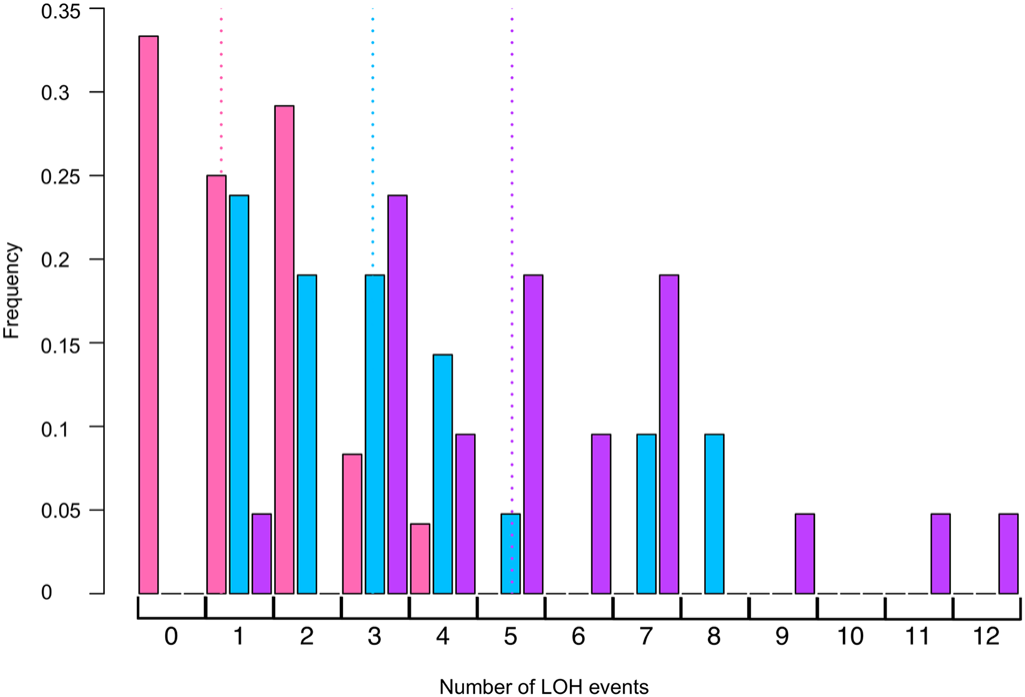
Histogram of numbers of unselected LOH events per colony induced by UV. Using SNP microarrays, we analyzed more than 20 colonies of each of three strains (r*ad14*, *rad14 mus81*, and *rad14 mms2*) for unselected LOH events induced by 1 J/m^2^. The numbers of LOH per colony are shown in this figure. The color code is: *rad14* (blue), *rad14 mus81* (pink), and *rad14 mms2* (purple). The dotted lines indicate the average number of LOH events per single colony using the same color code.

Double mutant *mus81 rad14* strains were very sensitive to UV. A dose of 1 J/m^2^, which reduced the viability of the *rad14* strain to 61% that of the untreated strain, lowered the viability of the double mutant strain to 0.2%. Consequently, we monitored the effect of UV in the double mutant strain on UV-induced mitotic recombination by analyzing single colonies formed from cells treated with 1 J/m^2^ rather than by measuring the frequency of red/white sectored colonies. The results of this analysis are shown in Table 4, and the distributions of the number of LOH events per colony for *rad14* and *rad14 mus81* strains are shown in Fig. 4. We found that the total unselected LOH events were significantly reduced in the double mutant compared to the single *rad14* mutant (p = 0.0005 by Mann-Whitney test). This result suggests that Mus81p is likely involved in generating recombinogenic DNA lesions at replication forks that are blocked at unexcised pyrimidine dimers; this interpretation will be further considered in the Discussion.

**Table 4:** Numbers of unselected UV-induced LOH events and other genomic changes in single colonies derived from *rad14*, *rad14 mus81*, *rad14 yen1*, and *rad14 mms2* diploids treated with 1 J/m^2^ of UV.

Strain name	Genotype	# Interstitial LOH events (ave. #/colony)	# Crossovers + BIR (ave. #/colony)	Total LOH events (ave. #/colony)	# Colonies analyzed	# Total interstitial in/dels (# *CUP1* alterations)	# Terminal in/dels	# Ploidy changes	Fold change in ave. # LOH events/colony relative to the *rad14* strain
YYy23.4	*rad14*	46 (2.2)	26 (1.2)	72 (3.4)	21	2 (1)	2^1^	1	1
YYy47.6	*rad14 mus81*	13 (0.5)^2^	17 (0.7)	30 (1.3)^3^	24	3 (0)	1	0	0.38
YYy83.8 YYy83.14	*rad14 yen1*	30 (1.7)	22 (1.2)	52 (2.9)	18	3 (3)	0	1	0.85
YYy69.10 YYy69.19	*rad14 mms2*	61 (2.9)	55 (2.6)^4^	116 (5.5)^4^	21	4 (3)	0	3	1.61

^1^One isolate also had a translocation.

^2^p<0.0001 compared to *rad14*.

^3^ p<0.001 compared to *rad14*.

^4^ p<0.01 compared to *rad14*.

We also examined the effects of the Yen1p HJ resolvase on the UV-induced recombination events in a *rad14* strain. After a dose of 15 J/m^2^ of UV, the single *yen1* mutant has about the same number of LOH events per cell as wild-type (Table 2). The double mutant *rad14 yen1* strain had approximately the same average number and types of LOH events per irradiated cells as the single *rad14* mutant (Table 4); none of the observed differences with wild-type were statistically significant. In summary, our analysis suggests that the Mus81p, but not the Yen1p, has a role in generating recombinogenic lesions in UV-treated *rad14* strains.

### Recombination between homologs induced by unexcised dimers is suppressed by the Mms2p-mediated PRR pathway

As discussed in the Introduction, unexcised pyrimidine dimers can be bypassed by either error-prone or error-free pathways [18]. The error-free pathway utilizes HR enzymes to catalyze either template switching between the two arms of the replication fork or fork regression. Since fork regression has not been observed for UV-treated strains, we assume that the primary pathway for error-free repair in our experiments is template switching. Template switching requires Ubc13p and Mms2p to poly-ubiquitinate PCNA. Although template switching is generally considered to be limited to the arms of the replication fork or to sister chromatids, most previous studies have been done in haploid strains. Consequently, we compared the frequency of UV-induced recombination in *rad14* and *rad14 mms2* diploids. If Mms2p is required for inter-homolog recombination, we would expect a reduction in recombination in the double mutant whereas, if Mms2p was required solely for inter-replication fork or inter-sister chromatid recombination, the double mutant should have elevated inter-homolog recombination.

The *rad14 mms2* strains, like the *rad14 mus81* strains were very UV sensitive. At a UV dose of 1 J/m^2^, the double mutant has 1% survival compared to 61% survival for *rad14* strain, and 0.2% survival for the *rad14 mus81* strain. Consequently, we compared the numbers and types of LOH events in single colonies derived from cells treated with 1J/m^2^ of UV (Table 4, Fig. 4). The average number of LOH events in a double mutant of *rad14 mms2* increased by 62% compared to the *rad14* strain; this difference is statistically significant (p = 0.008 by Mann-Whitney test). The main class of events that is elevated in the double mutant is the CO/BIR category (p = 0.001 by Mann-Whitney test). These results suggest that Mms2p promotes recombination between the arms of the replication fork and/or sister chromatids and, in the absence of Mms2p, the recombination intermediates are directed into an inter-homolog pathway of recombination.

### Lengths of UV-induced gene conversion tracts in wild-type, *rad14*, *mus81*, and *yen1* strains

In our previous analysis of the lengths of UV-induced mitotic gene conversion tracts in wild-type strains treated with 15 J/m^2^, we found a median length of 6.4 kb for all conversion tracts; median lengths of crossover-associated tracts (CO events) were longer than for conversions unassociated with crossovers (NCO events), 8.2 kb and 5.7 kb, respectively [4]. The median lengths of conversion events (CO plus NCO) in *rad14*, *mus81*, and *yen1* are all between 5.2 and 7.9 kb (Table 5). These lengths are not significantly different from the wild-type strain. As observed for the wild-type strain, the median lengths for the CO conversions exceed those for the NCO conversions for all strains. When we compared the lengths of conversions in NCO and CO categories, the only significant difference was that the lengths of CO events in the *mus81* strain significantly exceeded those of the wild-type strain irradiated with 15 J/m^2^ (p = 0.027 by Mann-Whitney test). One interpretation is that failure to cleave nicked HJs in the *mus81* mutants results in their maturation to double Holliday junctions that are associated with longer heteroduplex regions.

**Table 5:** Median lengths of gene conversion tracts in UV-induced LOH events in wild-type, *rad14*, *mus81*, and *yen1* strains.^1^

Strain name	Genotype	UV dose (J/m^2^)	Length of NCO tracts in kb (95% CL)	Length of CO tracts in kb (95% CL)	Length of all tracts in kb (95% CL)	# NCO conversions	# CO conversions	Total # conversions
PG311^2^	WT	1	ND^3^	4.3 (2.3–8.2)	ND^3^	ND	46	ND
YYy23.4	*rad14*	1	4.3 (2.8–5.2)	8.7 (5.9–11)	5.2 (3.8–7.7)	47	30	77
PG311^2^	WT	15	5.7 (4.5–6.6)	8.2 (6.6–10)	6.4 (5.8–7.3)	141	68	209
YYy76.14	*mus81*	15	6.8 (5.8–10.4)	16.9 (4.5–24)^4^	7.9 (5.9–12)	54	14	68
YYy71.28 YYy71.48	*yen1*	15	4.8 (3.7–6.3)	10.2 (8.3–12.6)	6.4 (5.2–9.7)	68	41	109

^1^For all experiments except PG311 treated with 1 J/m^2^, we examined conversion tract lengths in both selected and unselected events in sectored colonies in which the *SUP4-o* marker was on chromosome V. This analysis was done only in sectored colonies.

^2^Data from Yin and Petes (2013).

^3^Since there were very few unselected events in wild-type cells irradiated with 1 J/m^2^, all of the conversion events were associated with the selected crossover on chromosome V.

^4^ p<0.05 compared to wild-type.

### Distribution of LOH events resulting from unexcised pyrimidine dimers

For crossovers, the DNA lesions that initiate recombination should be located near the transitions between heterozygous and homozygous regions (the breakpoints of the LOH events). For gene conversions unassociated with crossovers, the initiating lesion should be located between the heterozygous sites flanking the conversion tract. Consequently, we determined whether these regions were significantly enriched for various types of chromosome elements such as replication origins, retrotransposons, palindromes, and regions associated with G4 motifs. The methods used for this analysis are described in the S1 Text, and the data are presented in S16 Table. We previously showed that spontaneous mitotic recombination events and LOH events induced by low levels of DNA polymerase *α* were enriched for replication-termination (TER) sequences [25] and other motifs associated with slow-moving or stalled replication forks [20,28]. In contrast, none of the chromosome elements tested were significantly over-represented among UV-induced LOH events in a wild-type strain [4].

We examined sixteen categories of chromosome elements for their representations at LOH breakpoints including: centromeres, tRNA genes, non-coding (nc) RNAs, solo long-terminal repeats (LTRs), early/middle ARS elements, late ARS elements, Rrm3p pause sites, palindromes, retrotransposons, G4 motifs, highly-transcribed and weakly-transcribed genes, TER sequences, regions with high levels of gamma-H2AX, snoRNAs and snRNAs, and genomic regions with inefficient TT dimer repair (references for the mapping of these elements are in S16 Table). In the *rad14* single mutant, none of these elements were significantly over- or under-represented. In contrast, in both the *mus81* and *rad14 mus81* strains, G4 (quadruplex) motifs are over-represented at the LOH breakpoints. In addition, in the *rad14 mus81* double mutant strain, we observed over-representation of retrotransposons (Ty elements), Rrm3p pause sites, regions with high levels of gamma H2AX, and regions associated with slow thymidine dimer repair (S16 Table).

Many of the over-represented motifs are associated with delayed or stalled replication forks in wild-type yeast cells, and are hotspots for mitotic recombination events induced by low levels of DNA polymerase *α* [28]. One explanation of our results is that the probability that a stalled replication fork will be broken is a function of at least three factors: certain DNA motifs (for example, quadruplex DNA) that cause replication forks to move slowly or stall, the presence of unexcised pyrimidine dimers near these motifs, and the presence or absence of Mus81p. Although Mus81p is required for about 60% of the LOH events induced by unexcised dimers, 40% are independent of Mus81p (Table 4). No significant associations were observed at the breakpoints of the *rad14 yen1* strain, although the *rad14 mms2* mutant had significant over-representations of ncRNAs and TER sequences.

### Analysis of spontaneous recombination events in *rad14*, *mus81*, and *yen1* single mutant strains

In strains with the *SUP4-o* marker near the right end of chromosome IV, we did a limited analysis of spontaneous recombination events derived from red/white sectored colonies in the *rad14*, *mus81*, and *yen1* strains. The primary purpose of this analysis was to ensure that none of the single mutants substantially elevated LOH in the absence of UV irradiation. The numbers of sectored colonies divided by the total number of colonies examined, the frequencies, and the strains names were: wild-type (55/1761664; 3.1x10^-5^; JSC25; [20]), *rad14* (20/144771; 1.4x10^-4^; YYy37.6/YYy37.8), *mus81* (10/277704; 3.6x10^-5^; YYy45.2/YYy46.1), and *yen1* (12/276315; 4.3x10^-5^; YYy72.22/YYy72.41). Although there is a statistically significant elevation of spontaneous crossovers in the *rad14* strain (as noted previously), the frequency of sectored colonies in unirradiated cells is two orders of magnitude less than in the irradiated cells. We also looked for sectored colonies derived from unirradiated cells of the *rad14 mus81* and *rad14 mms2* genotypes that had the *SUP4-o* marker on the left end of chromosome V. No sectored colonies were observed in colony totals of 22797 (*rad14*), 39466 (*rad14 mus81*), and 37493 (*rad14 mms2*). Although this analysis does not allow an accurate measurement of the sectoring frequency, we estimate that the frequency of spontaneous sector formation is less than 4x10^-5^ for all three strains. Thus, the spontaneous events do not contribute significantly to our estimates of the frequencies or types of UV-induced exchanges.

## Discussion

We performed a genome-wide analysis of inter-homolog recombination events induced by unexcised pyrimidine dimers, and we monitored the effect of various DNA repair enzymes on the frequency and types of these recombination events. The main conclusions from our study are: 1) unexcised pyrimidine dimers induced by irradiation of G1-synchonized cells strongly induce mitotic recombination between homologs, 2) most of the induced events reflect the repair of a single broken sister chromatid, 3) the recombinogenic effects of unexcised dimers are largely dependent on the Mus81p resolvase while in the NER-proficient strains, only crossovers are reduced by deletion of *MUS81*, and 4) *rad14 mms2* strains have elevated levels of UV-induced recombination events relative to the *rad14* single mutant, consistent with the hypothesis that the Mms2p-mediated PRR pathway channels DNA lesions for repair to the sister chromatid.

### Differences in UV-induced recombination between NER-proficient and NER-deficient cells

Although the recombinogenic effects of UV irradiation in yeast have been known for a long time [5], the nature of the recombinogenic DNA lesion and the pathways of lesion repair are still unclear. Based on the effects of UV irradiation in synchronized cells, Galli and Schiestl [2] suggested that NER-generated lesions (single-stranded nicks or gaps) could be converted into broken chromatids during DNA replication. Alternatively, the blocking of replication forks by unexcised dimers [10] could be converted into a recombinogenic DSB. One approach to distinguishing these possibilities is to compare UV-induced recombination events in wild-type and NER-deficient strains. Using this approach, Kadyk and Hartwell [6] found that wild-type diploid strains synchronized in G1 and treated with 30 J/m^2^ of UV had about a five-fold elevation in inter-homolog gene conversion and a two-fold induction in sister-chromatid recombination. In contrast, an NER-deficient *rad1* diploid strain irradiated with 1 J/m^2^ (approximately equivalently genotoxic with a dose of 30 J/m^2^ in a wild-type strain) had a six-fold elevation in sister-chromatid recombination and a three-fold reduction in the frequency of inter-homolog conversion events. From this analysis, they concluded that inter-homolog recombination was likely a consequence of nicks and gaps formed by NER, whereas unexcised dimers primarily induced sister-chromatid recombination rather than inter-homolog recombination.

In our study, UV treatment very strongly (>100-fold) stimulated mitotic crossovers (Table 1), as well as other types of recombination events (Table 2), in an NER-deficient (*rad14*) strain. There are several explanations for the discrepancy between our results and those of Kadyk and Hartwell [6]. First, different mutations were used to inactivate NER, *rad14* in our experiments and *rad1* in those of Kadyk and Hartwell. Rad1p, unlike Rad14p, is needed to process certain types of mitotic recombination intermediates [29] in addition to its requirement for NER. However, since Rad1p is not required for I-SceI-induced recombination events between homologs [7], lack of Rad1p would not be expected to reduce the frequency of UV-induced inter-homolog exchange. In addition, in the *rad14* (strain YYy23.4) and *rad1* (strain YYy327.1) cells treated with 1 J/m^2^ of UV, the frequencies of survival (49% and 47%, respectively), and red/white sectored colonies (8.1 x 10^-3^ and 6 x 10^-3^, respectively) were similar in the two strains.

A more likely explanation is the nature of the genetic system used to detect inter-homolog exchange in the two studies. In the Kadyk and Hartwell study [6], inter-homolog conversion events were detected by measuring the frequency of leucine prototrophs derived from a diploid with *leu1* heteroalleles. This system likely detects a very small fraction of the induced events. Assuming that mitotic conversion is a consequence of heteroduplex formation followed by DNA mismatch repair, a prototroph will be detected in only two situations: 1) the heteroduplex includes only one of the two *leu1* mutations or 2) the heteroduplex includes both mutations but the resulting repair event using different strands as the repair template (“patchy” repair). However, mitotic conversion events are usually longer than one gene (> 4 kb) and multiple mismatches within a heteroduplex are usually repaired using the same strand as a template [4,20]. In our analysis, we examine inter-homolog recombination events by a sectoring assay for crossovers or by detecting unselected crossovers and conversions using SNP microarrays. These systems have the advantages of having fewer constraints on the detection of gene conversion events and of being less selective as to the location of the events.

Although our assay is designed primarily to detect LOH events occurring between homologs, we recovered four interstitial deletions in the UV-treated *rad14* strains (Class R in S3 Table). Three of four deletions had directly-oriented Ty elements at the breakpoints of the deletion, and the fourth had one Ty element and a directly-oriented delta (LTR) (S4 Table). Thus, these deletions are likely generated by HR between the direct repeats rather than non-homologous end-joining. We have detected similar events previously in other genetically-unstable yeast strains [28,30]. The deletion events can be explained as intrachromatid “pop-outs”, unequal sister-chromatid crossovers, single-strand annealing, gene conversion, or off-set BIR events between sister chromatids. Some of these possibilities are shown in S4 Fig. Although deletions are observed in UV-treated *rad14* strains, none were observed in UV-treated wild-type strains. The reason for this difference is not understood although it could be related to the nature of the DNA lesion, or the kinetics of the repair process.

It should be emphasized that our system (and almost all genetic systems) are capable of detecting inter-homolog and unequal sister chromatid recombination events but cannot detect sister chromatid exchanges between perfectly-aligned chromatids. A UV dose of 1 J/m^2^ is expected to introduce about 500 pyrimidine dimers/diploid genome [31]. Since we detect about seven LOH events/sectored colony in the *rad14* strain (Table 2), most of the UV lesions must be bypassed by mechanisms that do not result in LOH, either recombination events that involve equal sister chromatid recombination or that utilize TLS polymerases.

### Nature of the recombinogenic DNA lesions in UV-treated yeast cells

As discussed above, one likely source of recombinogenic lesions in G1-synchronized wild-type cells treated with UV is nicks or gaps generated by NER; replication of such molecules would lead to DSBs [2]. If this lesion was solely responsible for initiating recombination in a wild-type cell, we would expect UV-irradiation of G1-synchronized cells to generate recombination events that indicate the break of a single chromatid (SCBs) (Fig. 2). In wild-type cells irradiated with 15 J/m^2^, however, we found that more than half of the events involved the repair of two sister chromatids broken at approximately the same position (DSCBs) [4]. Our interpretation of this result was that high doses of UV in G1-synchronized cells resulted in DSBs, and this interpretation was supported by physical evidence of a low frequency of UV-induced DSBs (5–10 DSBs in cells irradiated with 40 J/m^2^; [32]). In G1-synchronized cells irradiated with low levels of UV (1 J/m^2^), most of the recombination events were SCBs [4]. These results argue that the DSBs likely reflect the excision of closely-opposed pyrimidine dimers that arise independently [4]. An analysis of UV-induced recombination in *exo1* cells treated with 15 J/m^2^ showed that Exo1p-expanded NER gaps were required for both SCB and DSCB events [33]. In addition, as described in [34], an analysis of UV-induced events in G2-synchronized haploid cells showed that single-stranded gaps stimulated recombination between sister chromatids. In summary, there are likely several types of recombinogenic lesions generated by UV in wild-type cells: G1-associated DSBs resulting from the repair of closely-opposed dimers, S/G2-associated DSBs resulting from replication of a DNA molecule with an NER-generated gap, and single-stranded gaps; Exo1p-expanded NER gaps contribute to the formation of DSBs in both G1 and S/G2.

Most (76%) of the recombination events induced by UV in G1-synchronized *rad14* cells are SCBs (Table 3). These recombination events cannot be related to NER-generated DNA lesions, and we suggest that the relevant lesion is produced by breaks that occur at replication forks stalled by unexcised pyrimidine dimers. Two of the important HJ or nicked HJ resolvases in yeast are Mus81p and Yen1p. Consistent with the observations that Mus81p is required for DSB formation at fragile sites [35] and at camptothecin-stalled replication forks in mammalian cells [36], we found that loss of Mus81p reduced the frequency of UV-induced LOH events in the *rad14* strain by about 60%. This reduction could be explained as a reduction in the level of recombinogenic DSBs or as less efficient processing of recombination intermediates to yield LOH events. Previously, Ho *et al*. [15] showed that *mus81* mutants had reduced frequencies of I-*Sce*I-induced mitotic crossovers but wild-type levels of induced conversions. Our observation that *rad14 mus81* mutants have reduced UV-induced gene conversion unassociated with crossovers relative to *rad14* (Table 3) argues that Mus81p is involved in generating DSBs, although it may also have a role in downstream recombination events. In comparisons of the frequency of gene conversion events in wild-type and *mus81* strains treated with 15 J/m^2^ of UV, we found that Mus81p was required for crossovers, but not conversions unassociated with crossovers (Table 2), as expected from previous results.

Loss of Yen1p had no detectable effect on the frequency of LOH events in UV-treated *rad14* strains. Based on the substrate preferences for Mus81p and Yen1p [8], this result argues that the relevant recombinogenic structure of the blocked replication fork is a nicked HJ rather a ligated HJ. It has been suggested that extensively-regressed replication forks resembled ligated HJs which should be processed by Yen1p [37]. Thus, the lack of effect of Yen1p on the frequency of UV-induced exchange is consistent with the observation of Lopes *et al*. that UV blocks replication forks without causing extensive fork regression. Our observations are also in agreement with other studies [15,38] that show that Mus81p is more important than Yen1p for the generation of crossovers in yeast. The role of Yen1p may be somewhat lesion-specific, however, since loss of Yen1p reduces the frequency of sister chromatid recombination of DSBs generated by replication of nicked template [39]. We also cannot rule out the possibility that the Yen1p is not present during the time of replication fork blockage [40].

### Mechanisms to bypass unrepaired pyrimidine dimers that do not involve inter-homolog recombination

In addition to Mus81p-mediated resolution of a blocked replication fork producing a DSB, several other pathways allowing bypass of unexcised dimers exist: an error-prone pathway utilizing TLS polymerases and an error-free pathway that likely involves template switching between the arms of the replication fork [18]. Lopes *et al*. [11] showed that long single-stranded regions accumulate at replication forks in UV-treated yeast cells, and suggested that these events reflecting an uncoupling of leading and lagging strand replication. Both nicks and gaps are recombinogenic [34,41], and Giannattasio *et al*. [42] suggested that most template switching events were initiated with single-stranded gaps. Error-free template switching requires Mms2p, which catalyzes the polyubiquitination of PCNA, and Rad5p, a protein that mediates D-loop extension [43,44]. The error-prone TLS pathway is in competition with the error-free pathway. Elimination of the TLS pathway results in elevated frequencies of UV-induced sister-chromatid exchange [34].

In order to determine whether mutations in the error-free pathway contributed to LOH events between homologs, we examined *rad14 mms2* diploid strains. The double mutant strains had significantly elevated frequencies of inter-homolog recombination relative to the single mutant *rad14* strain (Table 4). Our preferred interpretation of this result is that Mms2p primarily promotes recombination between the arms of the replication fork and/or between sister chromatids rather than between homologs. Thus, a reduction in the frequency of recombination between sisters would result in an elevation in the frequency of inter-homolog interactions. This observation, as well as those of others, are consistent with a model in which Mus81p is primarily involved in producing DSBs by resolution of blocked replication forks, whereas Mms2p is mainly concerned with promoting recombination intermediates that involve a single-stranded gap.

**Fig 5:**
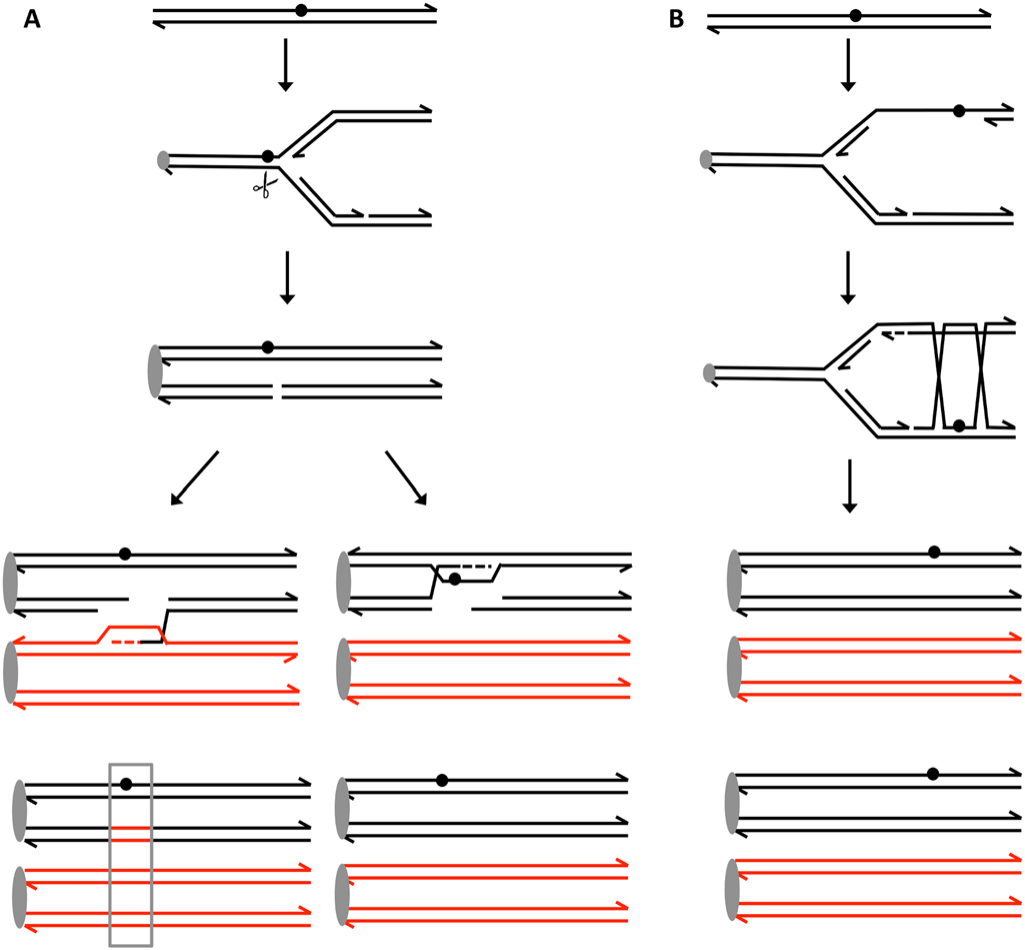
Recombination events induced by persistent UV damage in NER-deficient yeast strains. UV treatment results in the formation of a pyrimidine dimer (circle). A. Generation of a recombinogenic DSB. We suggest that the dimer-associated replication fork block can be converted into a DSB by the structure-nuclease Mus81p. The resulting DSB can be repaired either by recombination with the homolog, resulting in a detectable LOH event (boxed in grey), or by recombination with the sister chromatid which does not lead to LOH. B. Error-free bypass of lesions. When the 3’ end on the leading strand encounters a dimer, it can invade the sister chromatid (template switch), and continue replication. This branch of the post-replication repair requires Mms2p. We think that this pathway mainly involves interaction between sister chromatids without causing LOH and suppresses recombination between homologs.

In conclusion, unexcised UV dimers are a potent inducer of inter-homolog recombination in yeast. About 60% of this type of recombination is dependent on the Mus81p resolvase. The error-free PRR pathway mediated by Mms2p suppresses recombination between homologs, likely by channeling replication-blocking lesions into recombination between sister chromatids instead of homologs. These conclusions are summarized in Fig. 5.

## Materials and Methods

### Yeast strains

All strains are diploids formed by mating two sequence-diverged haploids W303–1A [45] and YJM789 [46]. The wild-type diploid (PG311) used in our analysis was described previously [21], and has the genotype: *MAT* **a** */MATα*::*natMX4 ade2–1/ade2–1 can1–100/can1*::*SUP4-o GAL2/gal2 his3–11*,*15/HIS3 leu2–3*,*112/LEU2 RAD5/RAD5 trp1–1/TRP1 ura3–1/ura3 V9229*::*hphMX4/V9229 V261553*::*LEU2/V261553*). The disruption of the *MATα* locus prevents sporulation of the diploid, and allows synchronization of the diploid in G1 using *α* pheromone. As described in the text, a crossover between the heterozygous *SUP4-o* marker and *CEN5* results in a red/white sectored colonies. The constructions of isogenic strains with homozygous mutations in *RAD14*, *MUS81*, *YEN1*, *MMS2*, and various double mutant strains are described in S17 Table.

In experiments to look at the rate of spontaneous crossovers, we used strains isogenic with JSC25 [20] in which the *SUP4-o* marker is inserted near the right telomere of chromosome IV. The genotype of JSC25 is: *MAT* **a** */MATα*::*hphMX4 ade2–1/ade2–1 can1–100*::*natMX4/CAN1*::*natMX4 GAL2/gal2 his3–11*,*15/HIS3 leu2–3*,*112/LEU2 RAD5/RAD5 trp1–1/TRP1 ura3–1/ura3 IV1510386*::*kanMX4-can1–100/IV1510386*::*SUP4-o*). Mutant strains isogenic to JSC25 with homozygous mutations in *RAD14*, *MU81*, and *YEN1* were constructed as described in S17 Table.

### Media and genetic methods

Standard media, genetic methods (mating, sporulation, transformation, and dissection), and DNA isolation procedures [47] were used unless otherwise indicated.

### Cell synchronization, and detection of spontaneous and UV-induced crossovers

All the UV experiments were done with cells synchronized in G1 with *α* pheromone as described previously [22]. After two hours of treatment with *α* pheromone, the cells were diluted, and plated on MAB6 solid medium (omission medium lacking arginine and containing 10 μg/ml adenine). The cells were immediately treated with 1 J/m^2^ or 15 J/m^2^ of UV using the TL-2000 UV Translinker. The plates were then wrapped in foil to prevent photoreversal of pyrimidine dimers, and incubated at 30°C. for two days. The plates were then incubated overnight at 4°C. This incubation period results in an intensification of the red color in red/white sectored colonies. To examine spontaneous crossovers, we allowed cells to form colonies by growing them on rich growth medium (YPD) for two days. Colonies were then diluted into water, plated onto MAB6 solid medium, and incubated for three days at room temperature. The plates were incubated at 4°C overnight as described above.

### SNP microarray analysis

The methods used for microarray analysis have been described in detail previously [3]. DNA from sectors or single colonies was isolated and sonicated using standard protocols. Experimental and control DNA samples were labeled with Cy5-dUTP and Cy3-dUTP, respectively, and hybridized in competition to Agilent-constructed SNP Microarrays. The whole-genome microarray was used to analyze all the UV-induced sectors and single colonies. The whole-genome microarray contains oligonucleotides that detect LOH for about 13,000 SNPs that differ between W303–1A and YJM789. Each SNP is represented by four 25-base oligonucleotides: two with sequences of the Watson and Crick strands of the W303–1A allele and two with sequences of the YJM789 allele. Following hybridization of the samples to the microarray, we scanned the array with GenePix scanner and GenePix Pro software. In-house R scripts were used for analyzing the microarrays. Based on the relative median hybridization ratio between experimental strain and control strain, which is heterozygous for all the SNPs, we determined which the SNPs were heterozygous or homozygous.

### Statistical analyses

The data were analyzed using chi-square, Mann-Whitney, or Fisher exact tests. Tests were performed with the VassarStat Website (http://vassarstats.net/), Excel, and R functions. Corrections for multiple comparisons were done using the method of Hochberg and Benjamini [48].

## Supporting Information

S1 Text Supplemental materials and methods, and discussion. (DOC)

S1 Fig Mitotic crossover induced in second division following irradiation. As in Fig. 2, the chromosomes of the hybrid diploid are indicated by black and red lines, with the black line showing the YJM789-derived homolog (the rectangle indicating *SUP4-o*) and the red line showing the W303–1A-derived homolog. Cells with zero, one and two copies of the *SUP4-o* suppressor form red, pink and white colonies, respectively. Ovals indicate centromeres. G1-synchronized cells were treated with UV. The UV-induced dimer (blue triangle) was not repaired in the first division, but segregated into one of the daughter cells (D2). In the subsequent cell division, the unrepaired dimer induced a reciprocal crossover, producing two granddaughter cells (GD2–1 and GD2–2) that subsequently gave rise to red and white sectors. The D1 daughter cell, which did not receive a UV-induced lesion, produced two granddaughter cells that gave rise to pink sectors. The net result of these events would be a tri-colored pink/red/white colony.
(TIF)

S2 Fig Patterns of LOH in sectored colonies consistent with a BIR event. In this figure, the homolog with the *SUP4-o* marker (solid black rectangle) and the unmarked homolog are shown as thick black and red lines, respectively. The thin lines indicate a different homolog. In red/white sectored colonies, a BIR event on the other homolog would have a terminal LOH event in one sector (D2) but not the other (D1).
(TIF)

S3 Fig A second-division crossover that mimics a BIR event. As in S2 Fig., the homologs with the selected event that generates the sectored colony are shown as thick lines, and a different homolog is shown with thin lines. We show a crossover on the selected homology occurring in the first division and a crossover on the unselected homolog occurring in the second division. Since we analyze only a single purified colony from the red and the white sectors, the LOH event on the unselected chromosome would appear to be the result of a BIR event rather than a crossover. For example, if colonies derived from GD1–1 and GD2–2 were analyzed, the LOH patterns would be identical to those observed for the BIR event depicted in S2 Fig.
(TIF)

S4 Fig Possible mechanisms for generating interstitial deletion or amplification events. Blue boxes depict direct repeats, and dotted lines represent intervening sequences between the repeats. Ovals indicate centromeres. We show all the four chromatids after replication. A. Deletion by intrachromatid “pop-out”. A heterozygous deletion is generated without a reciprocal duplication event. Note that we cannot distinguish this mechanism from single-strand annealing (SSA) or replication slippage. B. Reciprocal deletion/amplification events generated by unequal sister chromatid exchange. C. Duplication by gene conversion. In this depiction, the broken ends generated by a DSB in one repeat on the red chromatid invade different repeats on the black chromatid. DNA synthesis, analogous to gap repair, would produce a non-reciprocal duplication.
(TIF)

S1 Table Classes of UV-induced LOH events (selected on chromosome V and unselected on other chromosomes) in twenty sectored colonies resulting from treating G1-synchronized wild-type cells with a UV dose of 15 J/m^2^. The depictions in this Fig are LOH patterns observed in red/white sectored colonies derived from the irradiated diploid PG311. The data are a sub-set of those previously described in Yin and Petes (2013). Following irradiation, the cells were grown at 300 in medium lacking canavanine. In these depictions, green, red, and black lines indicate heterozygosity, homozygosity for the W303–1A-derived SNPs, and homozygosity for the YJM789-derived SNPs. Each pair of lines represents the LOH patterns in the two sectors. The lower case letters above and below the paired lines represent the transitions between heterozygous and homozygous SNPs or between two different regions of homozygous SNPs. The SGD coordinates for each transition are in S2 Table. The thin red line for Class Q1 indicates that the red sector was monosomic, retaining the W303–1A-derived chromosome. The sizes of the gene conversion tracts are not drawn to scale. The numbers of events of each type are shown in the right hand column. The abbreviation "SCB" indicates that the LOH event likely reflects the repair of a single chromatid break, and "DSCB" indicates that the LOH event is a consequence of the repair of two sister chromatids broken at same position (Yin and Petes, 2013). For some chromosomes, widely-separated LOH events were observed (Class L and Class P). If the LOH events were separated by a distance greater than the median conversion tract length plus two median absolute deviation (calculated in R), they were treated as two events. Interpretation for the classification of events is in the S1 Text in Yin and Petes 2013.
(XLS)

S2 Table SGD coordinates for transitions of LOH events (selected on chromosome V and unselected on other chromosomes) in sectored colonies treated with a UV dose of 15 J/m^2^ in G1-synchronized wild-type cells (strain PG311). This table shows the SGD coordinates of the transitions described in S1 Table for LOH events induced by 15 J/m2 in G1-synchronized cells of PG311. Each transition is represented by a pair of coordinates. The SGD coordinates used in this table are those of the 2010 version of SGD.
(XLS)

S3 Table Classes of UV-induced LOH events (selected on chromosome V and unselected on the other chromosomes) in sectored colonies resulting from treating G1-synchronized *rad14* cells with a UV dose of 1 J/m^2^. In this table, we include depictions of different classes of LOH events derived from twelve sectored colonies of YYy23.4. These colonies were derived from cells irradiated with 1 J/m2 of UV. The depictions follow the same code used in S1 Table. The strains of Class R have heterozygous deletions.
(XLS)

S4 Table SGD coordinates for transitions in sectored colonies induced by a UV dose of 1 J/m^2^ in G1-synchronized *rad14* cells. This table shows SGD coordinates for the LOH events depicted in S3 Table. The Class R events are deletions. All four have directly-oriented repeated elements at their breakpoints. The specific repeated elements for these four events are: 3RW (*YMRCTy1–3* and *YMRCTy1–4*), 32RW (*YDRWdelta10* and *YDRWTy2–2/YDRCTy1–2*), 152R/W (*YERCTy1–1* and *YERCTy1–2*), and 160RW (FS1 and FS2; these Ty elements are described in Lemoine *et al*., 2005).
(XLS)

S5 Table Classes of UV-induced LOH events (selected on chromosome V and unselected on other chromosomes) in sectored colonies resulting from treating G1-synchronized *mus81* cells with a UV dose of 15 J/m^2^. This table depicts UV-induced LOH events in the *mus81* strain YYy76.14. The depiction follow the same code used in S1 Table. The Class Q3 strain is trisomic for chromosome V.
(XLS)

S6 Table SGD coordinates for transitions in sectored colonies induced by a UV dose of 15 J/m^2^ in G1-synchronized *mus81* cells. This table has the SGD coordinates for the transitions shown in S1 Table (strain YYy76.14).
(XLS)

S7 Table Classes of UV-induced LOH events (selected on chromosome V and unselected on other chromosomes) in sectored colonies resulting from treating G1-synchronized *yen1* cells with a UV dose of 15 J/m^2^. This table shows the LOH events associated with UV treatment of the isogenic *yen1* strains YYy71.28 and YYy71.48. Note that in Class L, double crossovers are overrepresented.
(XLS)

S8 Table SGD coordinates for transitions in sectored colonies induced by a UV dose of 15 J/m^2^ in G1-synchronized *yen1* cells. In this table, we show the SGD coordinates for transitions of the UV-induced LOH events in the *yen1* strains YYy71.28 and YYy71.48. In this table, the sector labels that begin with 28- and 48- indicate that the sectors are derived from YYy71.28 and YYy71.48, respectively.
(XLS)

S9 Table Classes of UV-induced LOH events (unselected throughout the genome) in single colonies resulting from treating G1-synchronized cells with a UV dose of 1 J/m^2^. In this table, we show the patterns of LOH and aneuploidy in single colonies derived from irradiated cells in the following strains: YYy23.4 (*rad14*), YYy47.6 (*rad14 mus81*), YYy69.10 and YYy69.19 (*rad14 mms2*), and YYy83.8 and YYy83.14 (*rad14 yen1*). Each colony is represented by a double-width single line, with deletions and duplications shown as single-width and triple-width lines, respectively.
(XLS)

S10 Table SGD coordinates for transitions in single colonies resulting from a UV dose of 1 J/m^2^ in G1-synchronized *rad14* cells. In this table, we show the SGD coordinates for the single-colony LOH events in the *rad14* strain YYy23.4. The leftmost column distinguishes the individual colonies (c1, c2, etc.). All 21 colonies examined had at least one LOH event. The Classes d2 and d3 highlighted in green have Ty elements at their breakpoints. Class d2 is a terminal deletion and Class d3 is a terminal duplication, both on chromosome XIII. The likely explanation of these chromosome aberrations is that a break occurred in a Ty element on the right arm of XIII (*YMRCTy1–4*) and all of the sequences located distal to this breakpoint were deleted. The Ty element at the broken end of the right arm then invaded a Ty element on the left arm (*YMRWTy1–2*) and copied the sequences located centromere-distal to this Ty element, creating the duplication. Such break-induced replication (BIR) events between Ty elements have been detected previously (McCulley and Petes, 2010). In the "Event class" column, the designations *"CUP1* del" indicates that the colony had a decrease in the number of repeats in the *CUP1* tandem array. "*CUP1* del" is not depicted in S9 Table.
(XLS)

S11 Table SGD coordinates for transitions in single colonies resulting from a UV dose of 1 J/m^2^ in G1-synchronized *rad14 mus81* cells. In this table, we show the SGD coordinates for the single-colony LOH events in the *rad14 mus81* strain YYy47.6. Note that several of the colonies (for example, c50 and c51) had no LOH events. Most of the Class e events do not have SGD coordinates (marked NA) because they represent changes in chromosome number.
(XLS)

S12 Table SGD coordinates for transitions in single colonies resulting from a UV dose of 1 J/m^2^ in G1-synchronized *rad14 mms2* cells. In this table, we show the SGD coordinates for LOH transitions in the *rad14 mms2* strains YYy69.10 and YYy69.19. In the leftmost column, data derived from YYy69.10 are indicated with "10-" and those derived from YYy69.19 are indicated with "19-." The designations "*CUP1* del" and "*CUP1* amp" indicates that the colony had a decrease or increase in the number of repeats in the *CUP1* tandem array.
(XLS)

S13 Table SGD coordinates for LOH transitions in single colonies resulting from the treatment of G1-synchronized *rad14 yen1* cells with a UV dose of 1 J/m^2^. In this table, we show the SGD coordinates for LOH transitions in the *rad14 yen1* strains YYy83.8 and YYy83.14. In the leftmost column, data derived from YYy83.8 are indicated with "8-" and those derived from YYy83.14 are indicated with "14-." In the "Event class" column, the designations "*CUP1* del" and "*CUP1* amp" indicate that the colony had a decrease or increase in the number of repeats in the *CUP1* tandem array.
(XLS)

S14 Table SGD coordinates of each chromosome that were examined by the SNP microarrays to analyze unselected LOH events in wild-type, *rad14*, *mus81* and *yen1* sectors. In this table, we show the starting and ending SGD coordinates used to analyze unselected LOH events in strains in which the selected event was on the left arm of chromosome V (S1–8 Table). For most of the chromosomes, regions within 10 kb of the telomere could not be examined because these regions are repeated in the genome.
(XLS)

S15 Table SGD coordinates of the regions of each chromosome that were examined by the SNP microarrays to analyze LOH events in single colonies of *rad14*, *rad14 mus81* and *rad14 mms2* and *rad14 yen1* strains. In this table, we show the starting and ending SGD coordinates used to analyze unselected LOH events in single colony isolates (S9–13 Table). As stated in S14 Table, regions within 10 kb of the telomere could not be examined because these regions are repeated in the genome.
(XLS)

S16 Table Analysis of over- or under-represented SGD-annotated genome elements, various DNA motifs, damage-related genome elements, and highly-/weakly-transcribed genes within UV-induced conversion tracts. In this table, we compare the observed number of various types of genetic elements within the UV-induced gene conversion tracts for various genotypes with the expected number assuming a random distribution. The details of this analysis are described in S1 Text. The statistical significance of each comparison was determined using a chi-square test. The p values shown in the rightmost column are uncorrected p values. We applied the test of Hochberg and Benjamini (1990) to correct for multiple comparisons. The p values for comparisons involving elements that are significantly over-represented are highlighted in orange, and the p value for a comparison in which the element was under-represented is shown in green. The numbers and locations of tRNA genes, snoRNAs/snRNAs, retrotransposons, centromeres, ncRNA genes, ARS elements, and solo LTRs were determined using the YeastMine function in SGD. We corrected these numbers to exclude any elements that were in regions not included within the microarrays (S14 Table and S15 Table). References for other elements are: palindromes greater than 16 bp (Lisnic *et al*., 2005), G4 motifs (Capra *et al*., 2010), highly- and weakly-transcribed genes (Nagalakshmi *et al*., 2008), Rrm3p pause sites (Azvolinsky *et al*., 2009), regions with high levels of gamma-H2AX (Szilard *et al*., 2010), replication fork termination regions (Fachinetti *et a*l., 2010), and regions with inefficient TT dimer repair (Teng *et al*., 2011).
(XLS)

S17 Table Strain genotypes and constructions. (XLS)
