# Supplementary material for: Recombination between Homologous Chromosomes Induced by Unrepaired UV-Generated DNA Damage Requires Mus81p and Is Suppressed by Mms2p
Source: PLoS Genet. 2015 Mar 4;11(3):e1005026. doi: 10.1371/journal.pgen.1005026 (PMC4349867; doi:10.1371/journal.pgen.1005026)
Supplement: S1 Text — (DOC) [file pgen.1005026.s001.doc]

**Supplemental Text:**

**Recombination between homologous chromosomes induced by unrepaired UV-generated DNA damage requires Mus81p and is suppressed by Mms2p**

Yi Yin and Thomas D. Petes

Department of Molecular Genetics and Microbiology and University Program in Genetics and Genomics, Duke University Medical Center, Durham, NC 27710, USA.

**Statistical analysis of genetic elements over- or under-represented at LOH breakpoints in strains treated with UV**

The methods that were used in our analysis were very similar to those described previously [1-3]. For each unselected LOH event, we calculated (as discussed below) a “window” likely to contain the site at which the LOH event was initiated. For each strain, we determined the sum of these windows (in kb) for all of the isolates examined, and the sum of all of the genomic sequences that was located outside of these windows. For this analysis, we excluded the genomic regions that contained the selected events (the left arm of V). We examined the windows for their representations of sixteen different chromosome elements (tRNA genes, centromeres, long-terminal repeats, palindromes, etc.). We then compared the numbers of elements within the LOH windows and the number of elements located outside of these windows to the numbers expected by chance. The expected numbers were based on the number of elements in the genome as well as the amount of sequences involved in the conversion tracts versus the total amount of genome sequences examined. The observed and expected numbers were compared by chi-square analysis. The p values obtained from these comparisons were corrected for multiple comparisons using the method of Hochberg and Benjamini (1990, [4]). A summary of this analysis is in Table S16.

*Determination of LOH “windows”*

The “windows” used for the analysis were different for different events. For sectored colonies with a simple crossover (no associated conversion), the window contained sequences between the closest heterozygous SNP and the closest homozygous SNP to the transition. For example, for the Class H1 event (Table S1) on chromosome X in the sectored colony 79R/W, the window was from SGD coordinate 176818 to 183593 (Table S2). For 3:1 crossovers associated with a conversion, each sector has one transition between LOH and heterozygosity, but the positions of the transitions were in different places (for example, Class H4 in Table S1). For this type of event, the window was sequences located between the heterozygous SNP located closest to the transition (the left marker of label “a”) in the top sector (labeled “1” in Table S1) and the homozygous SNP closest to the transition (the right marker of label “b”) in the bottom sector (labeled “2” in Table S1). For example, for the Class H4 event on chromosome II in sector 8RW, the window is from SGD coordinates 442290 to 452926. Lastly, for an interstitial LOH event (for example, Class A2 in Table S1), we used the coordinates of the heterozygous SNPs that most closely flank the LOH event. For example, the Class A2 event on chromosome IV in sector 8RW (Table S2) has a window extending from SGD coordinates 624148 to 634749.

The windows for unsectored single colonies were determined using a similar procedure. For interstitial LOH events, we used the SGD coordinates of the heterozygous SNPs that most closely flank the LOH event. For terminal LOH events, we used the SGD coordinate of the closest heterozygous SNP to the transition and the coordinate of the closest homozygous SNP to the transition. For example, for Class b1 (Table S9) of single colony c3, the window for the event on chromosome VI is between 28650 and 31472.

*Genetic elements analyzed*

We examined 16 genetic elements to determine whether they were over- or under-represented in the LOH windows. The numbers and locations of tRNA genes, snoRNAs/snRNAs, retrotransposons, centromeres, ncRNA genes, ARS elements, and solo LTRs were downloaded from YeastMine in SGD. The “intersect” function in “BEDTools” [5] is used for determining the number of the genome elements that overlap our gene conversion tracts. We corrected these numbers to exclude any elements that were in regions not included within the microarrays (Tables S14 and S15). References for other elements are: palindromes greater than 16 bp [6], G4 motifs [7], highly- and weakly-transcribed genes [8], Rrm3p pause sites [9], regions with high levels of gamma-H2AX [10], replication fork termination regions [11], and regions with inefficient TT dimer repair [12]. For the analysis of the effects of high or low levels of transcription, we determined whether the 5% most highly-transcribed or the 5% most weakly-transcribed genes were over-represented in the LOH breakpoints. The analysis is summarized in Table S16.

Additional references mentioned in the Tables S1 to S17 are listed [13-20].

**References**

1. Song W, Dominska M, Greenwell PW, Petes TD (2014) Genome-wide high-resolution mapping of chromosome fragile sites in Saccharomyces cerevisiae. Proc Natl Acad Sci U S A.

2. St Charles J, Petes TD (2013) High-resolution mapping of spontaneous mitotic recombination hotspots on the 1.1 Mb arm of yeast chromosome IV. PLoS Genet 9: e1003434.

3. Yin Y, Petes TD (2013) Genome-wide high-resolution mapping of UV-induced mitotic recombination events in Saccharomyces cerevisiae. PLoS Genet 9: e1003894.

4. Hochberg Y, Benjamini Y (1990) More powerful procedures for multiple significance testing. Stat Med 9: 811-818.

5. Quinlan AR, Hall IM (2010) BEDTools: a flexible suite of utilities for comparing genomic features. Bioinformatics 26: 841-842.

6. Lisnic B, Svetec IK, Saric H, Nikolic I, Zgaga Z (2005) Palindrome content of the yeast Saccharomyces cerevisiae genome. Curr Genet 47: 289-297.

7. Capra JA, Paeschke K, Singh M, Zakian VA (2010) G-quadruplex DNA sequences are evolutionarily conserved and associated with distinct genomic features in Saccharomyces cerevisiae. PLoS Comput Biol 6: e1000861.

8. Nagalakshmi U, Wang Z, Waern K, Shou C, Raha D, et al. (2008) The transcriptional landscape of the yeast genome defined by RNA sequencing. Science 320: 1344-1349.

9. Azvolinsky A, Giresi PG, Lieb JD, Zakian VA (2009) Highly transcribed RNA polymerase II genes are impediments to replication fork progression in Saccharomyces cerevisiae. Mol Cell 34: 722-734.

10. Szilard RK, Jacques PE, Laramee L, Cheng B, Galicia S, et al. (2010) Systematic identification of fragile sites via genome-wide location analysis of gamma-H2AX. Nat Struct Mol Biol 17: 299-305.

11. Fachinetti D, Bermejo R, Cocito A, Minardi S, Katou Y, et al. (2010) Replication termination at eukaryotic chromosomes is mediated by Top2 and occurs at genomic loci containing pausing elements. Mol Cell 39: 595-605.

12. Teng Y, Bennett M, Evans KE, Zhuang-Jackson H, Higgs A, et al. (2011) A novel method for the genome-wide high resolution analysis of DNA damage. Nucleic Acids Res 39: e10.

13. Barbera MA, Petes TD (2006) Selection and analysis of spontaneous reciprocal mitotic cross-overs in Saccharomyces cerevisiae. Proc Natl Acad Sci U S A 103: 12819-12824.

14. Casper AM, Greenwell PW, Tang W, Petes TD (2009) Chromosome aberrations resulting from double-strand DNA breaks at a naturally occurring yeast fragile site composed of inverted ty elements are independent of Mre11p and Sae2p. Genetics 183: 423-439, 421SI-426SI.

15. Lee PS, Greenwell PW, Dominska M, Gawel M, Hamilton M, et al. (2009) A fine-structure map of spontaneous mitotic crossovers in the yeast Saccharomyces cerevisiae. PLoS Genet 5: e1000410.

16. Lemoine FJ, Degtyareva NP, Lobachev K, Petes TD (2005) Chromosomal translocations in yeast induced by low levels of DNA polymerase a model for chromosome fragile sites. Cell 120: 587-598.

17. Longtine MS, McKenzie A, 3rd, Demarini DJ, Shah NG, Wach A, et al. (1998) Additional modules for versatile and economical PCR-based gene deletion and modification in Saccharomyces cerevisiae. Yeast 14: 953-961.

18. McCulley JL, Petes TD (2010) Chromosome rearrangements and aneuploidy in yeast strains lacking both Tel1p and Mec1p reflect deficiencies in two different mechanisms. Proc Natl Acad Sci U S A 107: 11465-11470.

19. Zhao X, Muller EG, Rothstein R (1998) A suppressor of two essential checkpoint genes identifies a novel protein that negatively affects dNTP pools. Mol Cell 2: 329-340.

20. Goldstein AL, McCusker JH (1999) Three new dominant drug resistance cassettes for gene disruption in Saccharomyces cerevisiae. Yeast 15: 1541-1553.
