# Supplementary figures and images for: Recombination between Homologous Chromosomes Induced by Unrepaired UV-Generated DNA Damage Requires Mus81p and Is Suppressed by Mms2p
Source: PLoS Genet. 2015 Mar 4;11(3):e1005026. doi: 10.1371/journal.pgen.1005026 (PMC4349867; doi:10.1371/journal.pgen.1005026)

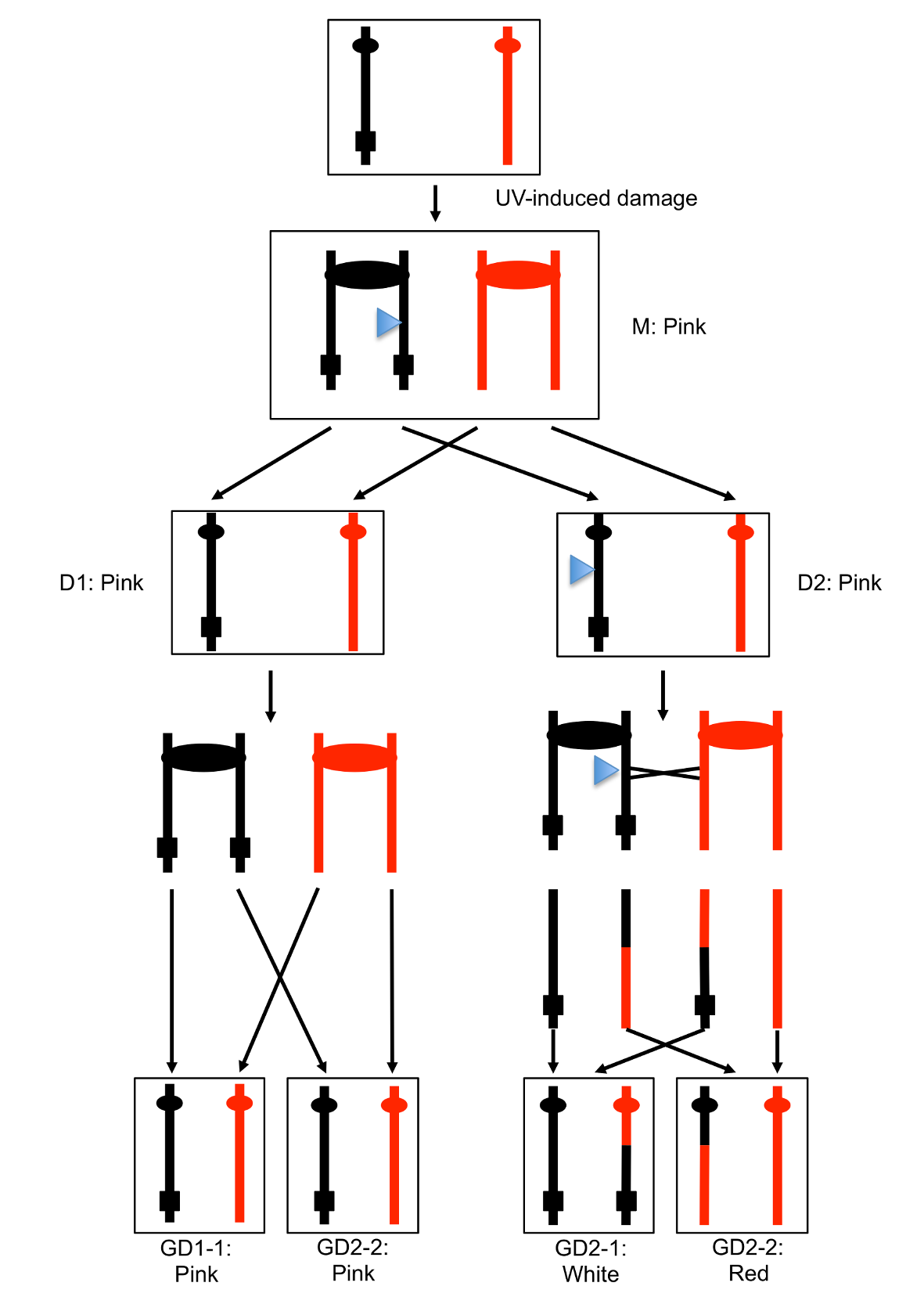

Supplement: S1 Fig — As in Fig. 2, the chromosomes of the hybrid diploid are indicated by black and red lines, with the black line showing the YJM789-derived homolog (the rectangle indicating SUP4-o) and the red line showing the W303–1A-derived homolog. Cells with zero, one and two copies of the SUP4-o suppressor form red, pink and white colonies, respectively. Ovals indicate centromeres. G1-synchronized cells were treated with UV. The UV-induced dimer (blue triangle) was not repaired in the first division, but segregated into one of the daughter cells (D2). In the subsequent cell division, the unrepaired dimer induced a reciprocal crossover, producing two granddaughter cells (GD2–1 and GD2–2) that subsequently gave rise to red and white sectors. The D1 daughter cell, which did not receive a UV-induced lesion, produced two granddaughter cells that gave rise to pink sectors. The net result of these events would be a tri-colored pink/red/white colony. (TIF) [file pgen.1005026.s002.tif]

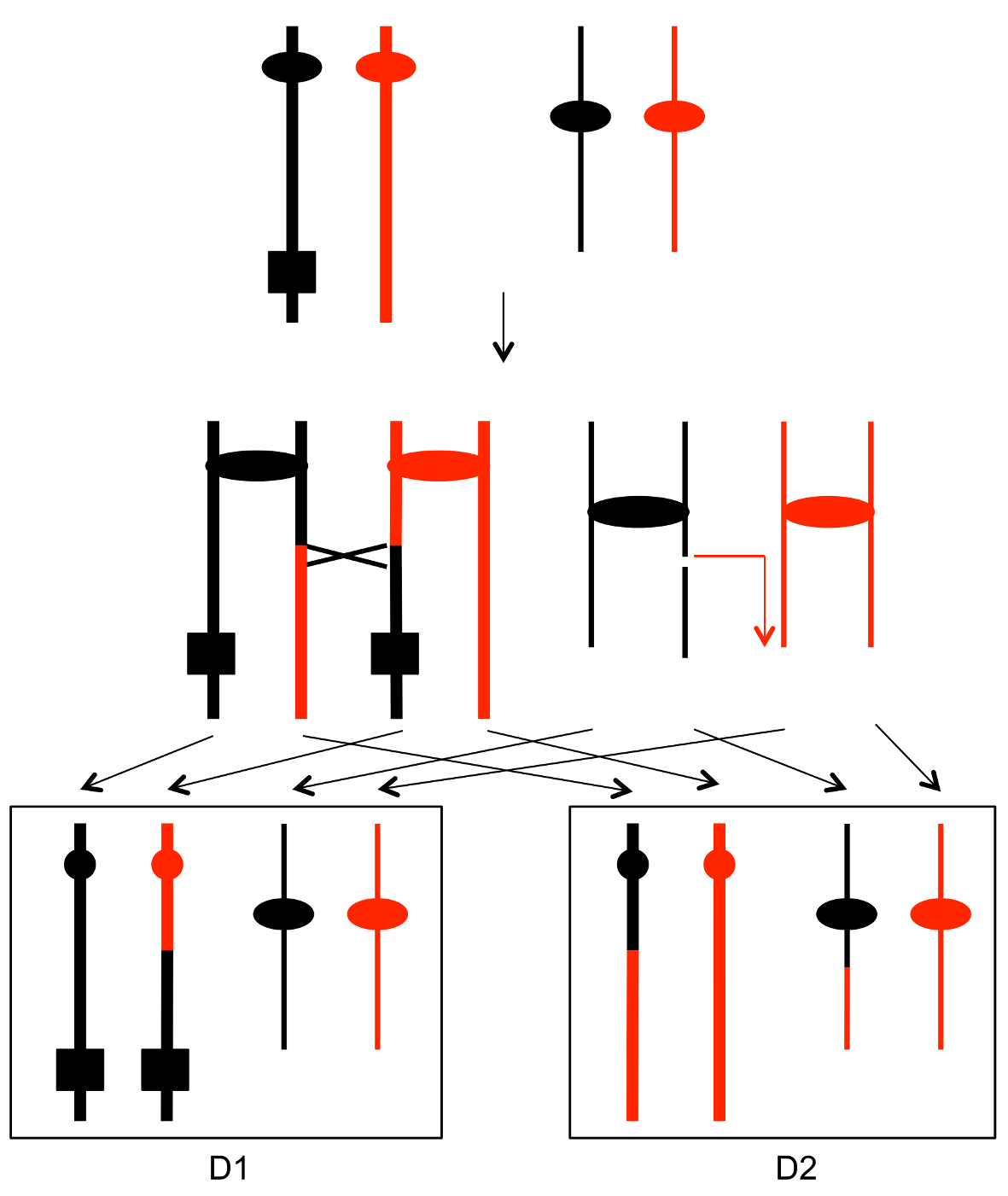

Supplement: S2 Fig — In this figure, the homolog with the SUP4-o marker (solid black rectangle) and the unmarked homolog are shown as thick black and red lines, respectively. The thin lines indicate a different homolog. In red/white sectored colonies, a BIR event on the other homolog would have a terminal LOH event in one sector (D2) but not the other (D1). (TIF) [file pgen.1005026.s003.tif]

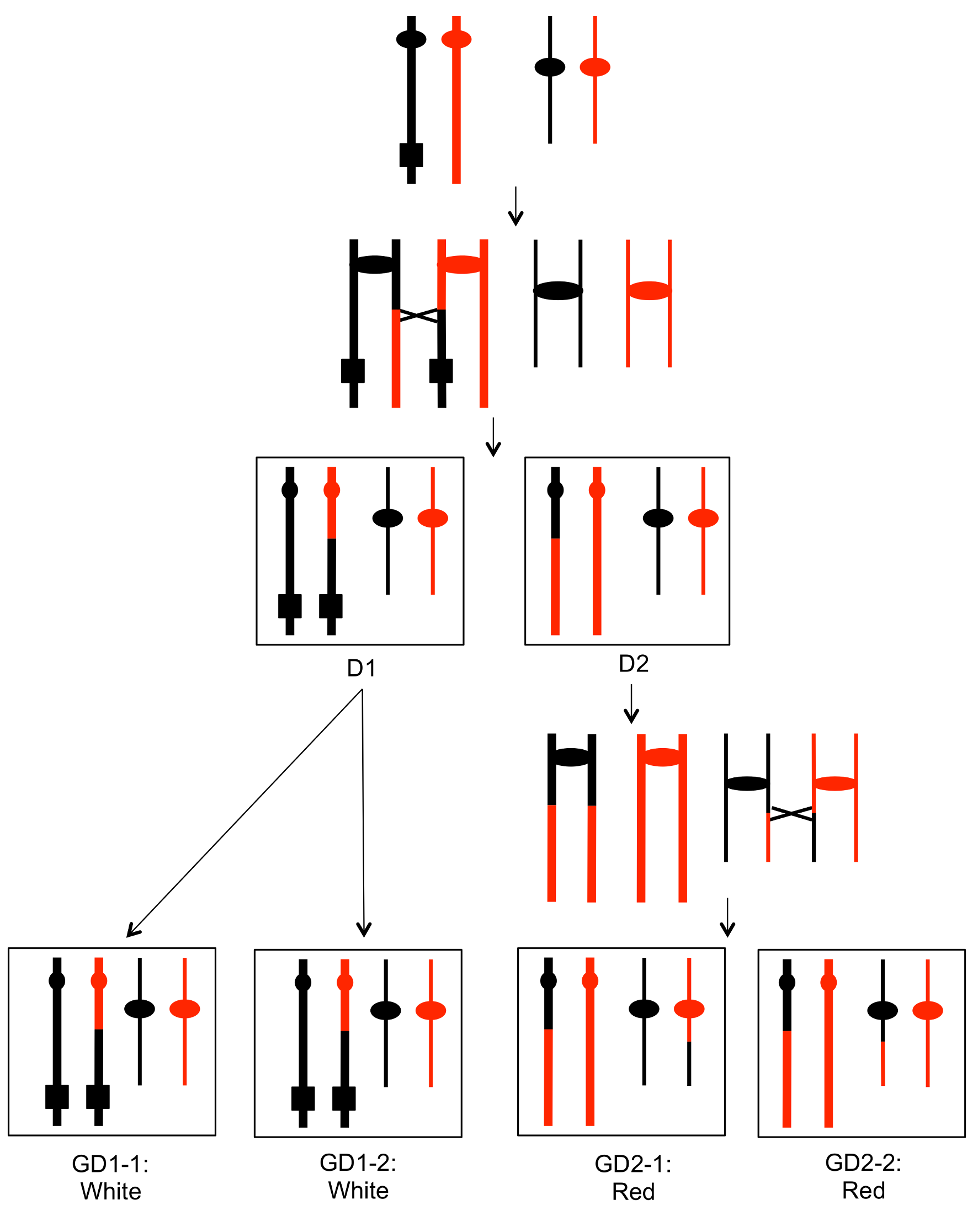

Supplement: S3 Fig — As in S2 Fig., the homologs with the selected event that generates the sectored colony are shown as thick lines, and a different homolog is shown with thin lines. We show a crossover on the selected homology occurring in the first division and a crossover on the unselected homolog occurring in the second division. Since we analyze only a single purified colony from the red and the white sectors, the LOH event on the unselected chromosome would appear to be the result of a BIR event rather than a crossover. For example, if colonies derived from GD1–1 and GD2–2 were analyzed, the LOH patterns would be identical to those observed for the BIR event depicted in S2 Fig. (TIF) [file pgen.1005026.s004.tif]

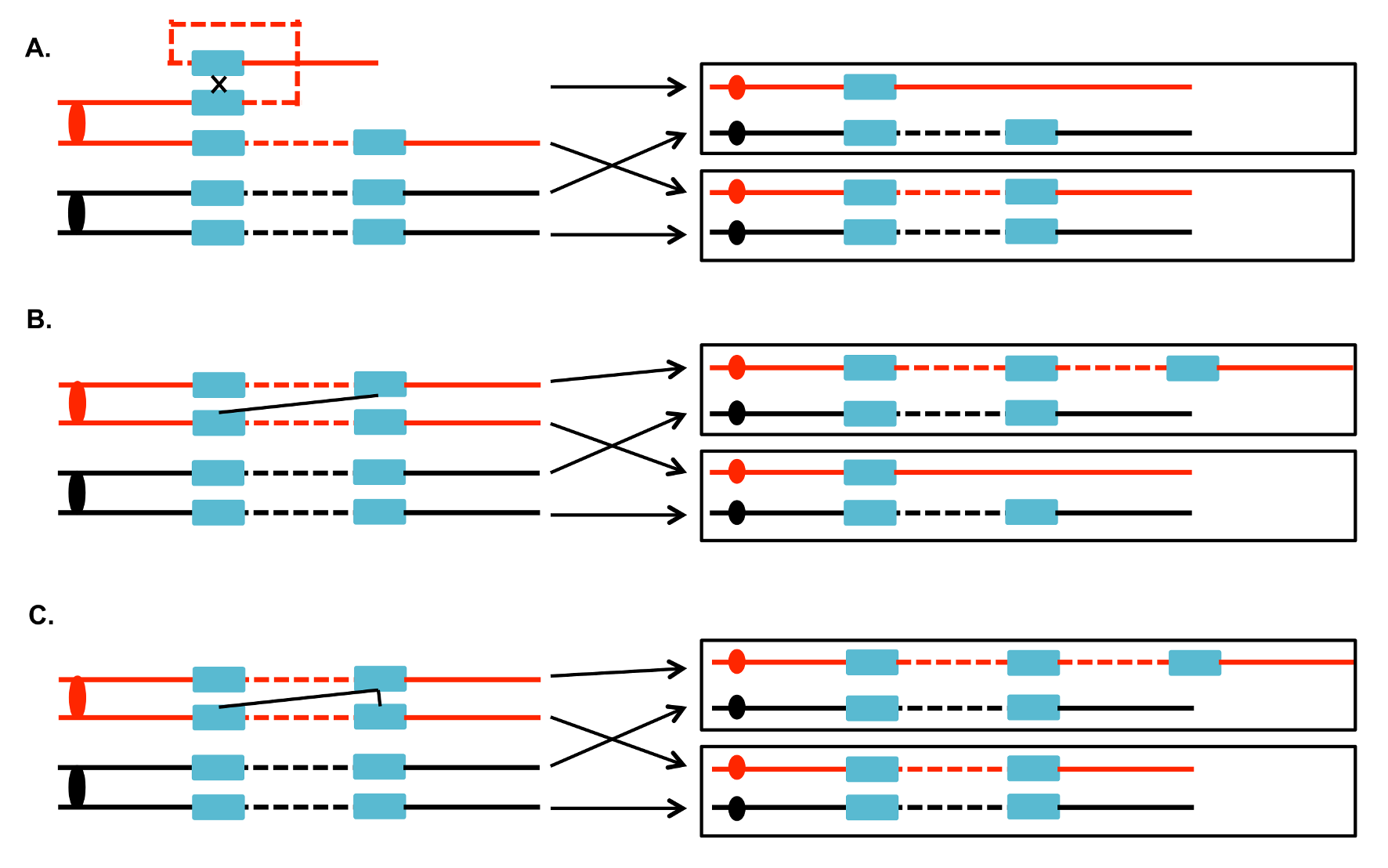

Supplement: S4 Fig — Blue boxes depict direct repeats, and dotted lines represent intervening sequences between the repeats. Ovals indicate centromeres. We show all the four chromatids after replication. A. Deletion by intrachromatid “pop-out”. A heterozygous deletion is generated without a reciprocal duplication event. Note that we cannot distinguish this mechanism from single-strand annealing (SSA) or replication slippage. B. Reciprocal deletion/amplification events generated by unequal sister chromatid exchange. C. Duplication by gene conversion. In this depiction, the broken ends generated by a DSB in one repeat on the red chromatid invade different repeats on the black chromatid. DNA synthesis, analogous to gap repair, would produce a non-reciprocal duplication. (TIF) [file pgen.1005026.s005.tif]
